# Structural and functional insights into the E3 ligase, RNF126

**DOI:** 10.1038/srep26433

**Published:** 2016-05-19

**Authors:** Ewelina M. Krysztofinska, Santiago Martínez-Lumbreras, Arjun Thapaliya, Nicola J. Evans, Stephen High, Rivka L. Isaacson

**Affiliations:** 1 Department of Chemistry, King’s College London, Britannia House, Trinity Street, London, SE1 1DB, UK; 2 Faculty of Life Sciences, University of Manchester, The Michael Smith Building, Oxford Road, Manchester, M13 9PT, UK

## Abstract

RNF126 is an E3 ubiquitin ligase that collaborates with the BAG6 sortase complex to ubiquitinate hydrophobic substrates in the cytoplasm that are destined for proteasomal recycling. Composed of a trimeric complex of BAG6, TRC35 and UBL4A the BAG6 sortase is also associated with SGTA, a co-chaperone from which it can obtain hydrophobic substrates. Here we solve the solution structure of the RNF126 zinc finger domain in complex with the BAG6 UBL domain. We also characterise an interaction between RNF126 and UBL4A and analyse the competition between SGTA and RNF126 for the N-terminal BAG6 binding site. This work sheds light on the sorting mechanism of the BAG6 complex and its accessory proteins which, together, decide the fate of stray hydrophobic proteins in the aqueous cytoplasm.

Eukaryotic cells have developed quality control mechanisms that sustain protein homeostasis by modulating protein folding, targeting and degradation. These mechanisms protect the cell from a range of physiological challenges including: stress-induced inhibition of protein synthesis; mutations in targeting signals; aberrant protein conformations and defective protein translocation into the ER and mitochondria^1^. Mislocalised membrane and secretory proteins represent a particular challenge because of the danger cytosolic exposure poses to their hydrophobic stretches. Hence, a failure of authentic protein targeting can result in the mislocalization of misfolded and aggregation-prone precursors to the cytosol^2^,^3^,^4^. Understanding how misfolded proteins are selected for degradation has implications in various diseases including cancer, cystic fibrosis and neurodegenerative disorders such as Parkinson’s disease and Alzheimer’s disease^5^,^6^.

A network of chaperones has evolved that can both aid refolding of misfolded proteins and/or promote their degradation via the ubiquitin–proteasome system^7^. The specificity of this proteolysis is commonly maintained by E3 ubiquitin–protein ligases, which select the appropriate substrate for ubiquitination through collaboration with various molecular chaperones involved in the binding and recognition of misfolded protein substrates^8^,^9^,^10^.

The heterotrimeric BAG6 complex, composed of BAG6 (BCL2-associated athanogene 6), TRC35 (transmembrane recognition complex 35) and UBL4A (ubiquitin-like protein 4A), together with the cochaperone SGTA (small, glutamine-rich, tetratricopeptide repeat-containing, protein alpha), participate in several protein homeostasis control mechanisms: tail-anchored (TA) protein targeting to the ER^11^,^12^, mislocalised protein degradation^13^ and ER-associated degradation^14^. They act by recognising the exposed hydrophobic regions of these different targets (TMDs of TA proteins and hydrophobic regions of mislocalised membrane and secretory proteins (MLPs)) and either facilitate their polyubiquitination and degradation at the proteasome^13^,^15^ or their correct onward delivery to the ER.

SGTA collaborates with the BAG6 complex to maintain hydrophobic substrates in non-ubiquitinated states and/or actively promotes their deubiquitination. SGTA competes with ubiquitination machinery for MLPs by binding their exposed hydrophobic degrons in the cytosol and rescuing them from degradation^16^,^17^. Hence, SGTA and the BAG6 complex are key players in MLP quality control and their collaborative work is vital in determining the fate of hydrophobic substrates. In the case of TA proteins it has even been suggested that the actions of SGTA may constitute a rescue pathway for substrates that are prematurely ubiquitinated^17^. Significantly, the combined activity of SGTA and the BAG6 complex is also implicated in the post-translational insertion of TA proteins into the membrane of the endoplasmic reticulum (ER)^11^,^18^. Hence, SGTA and the BAG6 complex engineer the transfer of newly-synthesised TA-proteins to the downstream targeting factor TRC40^19^. TRC40 recognizes hydrophobic TA regions^20^ and promotes their membrane insertion at the ER via a cognate receptor comprising the WRB and CAML proteins^21^,^22^. Likewise, the BAG6 complex and SGTA have been linked with the pathway for endoplasmic reticulum-associated degradation (ERAD) where they promote the efficient removal of misfolded polypeptides from the ER and maintain client solubility in the cytosol until delivery to the proteasome^23^,^24^.

Recent studies identified RNF126 as a soluble E3 ligase that contributes to BAG6-mediated quality control^4^. BAG6 recognises MLPs and recruits RNF126 for the ubiquitination of these hydrophobic clients that are destined for proteasomal degradation. RNF126 belongs to the family of RING (really interesting new gene) E3 ligases and contains two distinct domains: an N-terminal zinc-finger domain (residues 1–100), and a C- terminal RING domain (residues 229–270)^25^ with the former region thought to play an important role in its interaction with the UBL domain of BAG6^4^. In addition to its quality control function, RNF126 has been implicated in the endosomal sorting of cell surface receptors (CI-MPR)^26^,^27^ and the degradation of p21 cyclin-dependent kinase inhibitor, thereby promoting cancer cell proliferation^25^. To date, full-length RNF126 has not been structurally characterised, although a solution structure of the mouse RING domain has been solved (PDB Accession Number: 2ECT; no associated publication).

In this study we present the solution structure of the RNF126_NZF (N-terminal zinc finger domain) as well as the structure of RNF126_NZF/BAG6_UBL complex and characterise the interaction of RNF126 with the UBL domains from both BAG6 and UBL4A using NMR (Nuclear Magnetic Resonance) spectroscopy, native gel electrophoresis, ITC (Isothermal Titration Calorimetry) and MST (Microscale Thermophoresis). We also analyse the competition between SGTA and RNF126 for the same binding site on the BAG6 protein, and use our findings to suggest a molecular model for BAG6/SGTA mediated quality control.

## Results

### NMR solution structure of the RNF126 N-terminal zinc finger

The N-terminal region of RNF126 (residues 1–100 previously defined as the BAG6-interacting module^4^) yields a ^1^H-^15^N HSQC spectrum indicative of a partially folded protein. Backbone assignment of residues 1–66 shows that residues 1–40 constitute the structured region while the remainder presents the chemical shift dispersion typical of disordered proteins. A new construct (residues 1–40, hereafter named RNF126_NZF) displays comparable spectra to the longer version, showing that the additional truncation has no effect on the folding of the zinc finger region (Figure S1).

**Figure 1:**
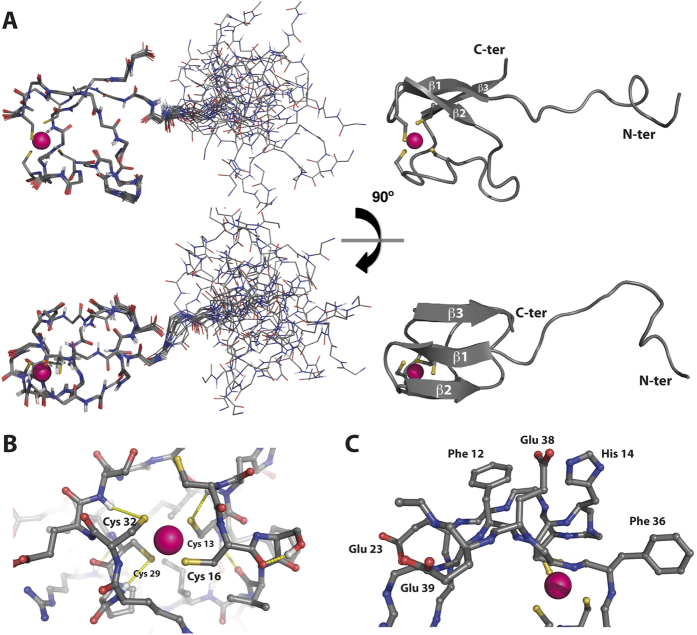
NMR solution structure of RNF126_NZF. (**A**) Orthogonal views of ensemble backbone and cartoon representations showing the 20 lowest energy ARIA-calculated structures as deposited in the PDB (Accession number: 2N9O). (**B**) Detailed view of the zinc finger coordination shell showing the cysteine residues coordinating the zinc cation; hydrogen bonds in the second coordination shell are shown as yellow dashed lines. (**C**) Detailed view of the solvent-exposed β-sheet interface. Polar and hydrophobic residues present at this interface are depicted using ball-and-stick representation. Carbon atoms are coloured in grey, oxygen atoms in red, nitrogen atoms in dark blue and sulphur atoms in yellow; zinc cation in magenta.

We acquired the complete battery of triple resonance experiments using the shorter construct, RNF126_NZF, to facilitate full backbone and side-chain assignment (BMRB Accession Number: 25913). We solved the solution structure using standard methods analogous to our previous approach^28^,^29^ (Fig. 1A; PDB Accession Number: 2N9O; statistical parameters in Table 1). The folded domain comprises residues Arginine 10 to Leucine 40. This region contains a three-stranded short antiparallel β-sheet (β1 = Y11-C13; β2 = V18-I20; β3 = I37-E39) and a zinc finger involving four cysteine residues; one pair from the β_1_-β_2_ short loop (C13; C16) and the other pair from the long structured region between strands β_2_ and β_3_ (C29; C32); we confirmed the equimolar presence of zinc cation by ICP/MS. The first zinc coordination shell contains the four cysteine side-chains in tetrahedral conformation (Fig. 1B) whose chemical shift values for ^13^Cα (~59 ppm) and ^13^Cβ (~31 ppm) are consistent with their zinc-binding character^30^. The second coordination shell is defined by the formation of three hydrogen bonds between the cysteine sulphur atoms (C13, C29 and C32) and the amide group of the residue at position +2 (C15, R31 and S34 respectively) (Fig. 1B); we summarise all zinc coordination parameters in Table S1. The long structured loop β_2_-β_3_ folds against one side of the β-sheet burying several non-polar residues and creating a stable hydrophobic core; by contrast the other side of the β-sheet is solvent exposed, containing some glutamic acid side-chains and aromatic rings and constituting a putative platform for protein-protein interaction (Fig. 1C).

### Biophysical characterisation of RNF126-BAG6 interaction

**Figure 2:**
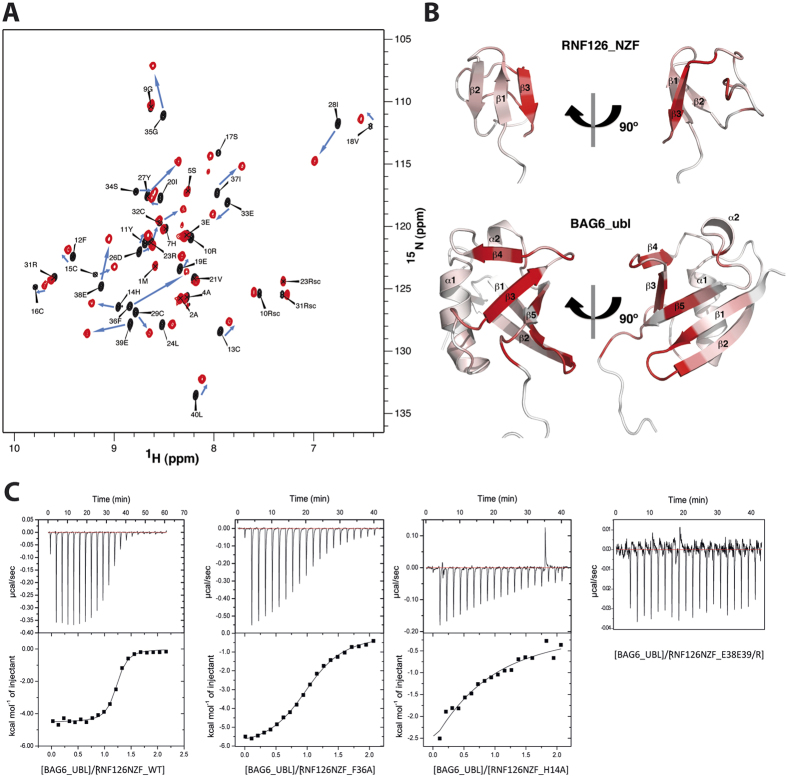
RNF126_NZF/BAG6_UBL interaction studies. (**A**) ^15^N-^1^H HSQC spectra of free RNF126_NZF (black, assigned peaks) and RNF126_NZF bound to BAG6_UBL (1:1 molar ratio in red). Blue arrows indicate the CSPs upon titration. “sc” labelling refers to side-chain assignments. (**B**) Orthogonal cartoon views of RNF126_NZF (this paper) and BAG6_UBL (PDB: 1WX9) coloured according to reciprocal CSPs with most perturbed residues shown in red. (**C**) ITC data showing binding of RNF126_NZF (wild type and mutants) to BAG6_UBL. Affinity constants, determined by ITC were: K_d_ of wild type RNF126_NZF = 0.40 ± 0.05 μM; K_d_ of F36A mutant = 5.0 ± 0.3 μM; K_d_ of H14A mutant = >50 μM and K_d_ of E38E39/R mutant could not be calculated.

To analyse the binding interface between RNF126 and BAG6, we carried out reciprocal chemical shift perturbation (CSP) studies by titrating unlabelled RNF126_NT (both 1–40 and 1–100 constructs) into ^15^N-labelled BAG6_UBL and vice versa. We used our backbone assignments described above in concert with the assignments for BAG6_UBL deposited in the BMRB (Accession Number: 11263). Both RNF126 constructs show comparable shifts upon BAG6_UBL binding and have the same effect on the BAG6_UBL spectrum confirming that residues 1–40 are necessary and sufficient for the interaction (Figure S2). We therefore adopted the 1–40 construct for all subsequent experiments. The spectra show binding in a slow exchange regimen (Figs 2A and S3A), suggesting a high-affinity complex with a saturation point close to a 1:1 molar ratio. The assignment of the bound state spectra showed that the β-sheet (predominantly the β_3_ strand) of RNF126_NZF is clearly affected upon titration, together with some residues from the β_2_-β_3_ loop. The binding region in BAG6_UBL is located along its exposed β-sheet; β_3_, β_4_, β_5_ strands, the loop between β_1_ and β_2_ and also includes part of the C-terminal region (Fig. 2B). In addition, we confirmed and characterised the binding between RNF126_NZF and BAG6_UBL by native gel electrophoresis, microscale thermophoresis (MST; Figure S4) and isothermal titration calorimetry (ITC) (Fig. 2C). ITC results indicate 1:1 stoichiometry with a dissociation constant of 0.40 ± 0.05 μM in agreement with the slow exchange regime of the NMR titration data. The favourable enthalpy and entropy values obtained from ITC (ΔH = −4.52 ± 0.04 kcal/mol; ΔS = 14.1 ± 0.4 cal/mol·K) suggest that the complex formation between RNF126 and BAG6 is driven by the establishment of both hydrogen bonds and hydrophobic interactions.

Finally to further characterize the interface, we designed some RNF126_NZF mutants based on the largest chemical shift perturbations (His14/Ala, Phe36/Ala and double mutant for charge inversion Glu38/Arg Glu39/Arg) and analysed their effect on BAG6_UBL interaction using ITC (Fig. 2C), native PAGE mobility shift assay and NMR (Figure S5). All mutations result in a drop in binding affinity of at least an order of magnitude, proving that they are involved in the interaction with BAG6. The wild-type structure is preserved in each mutant, as judged by 1D NMR (Figure S5).

### RNF126_NZF/BAG6_UBL complex structure

**Figure 3:**
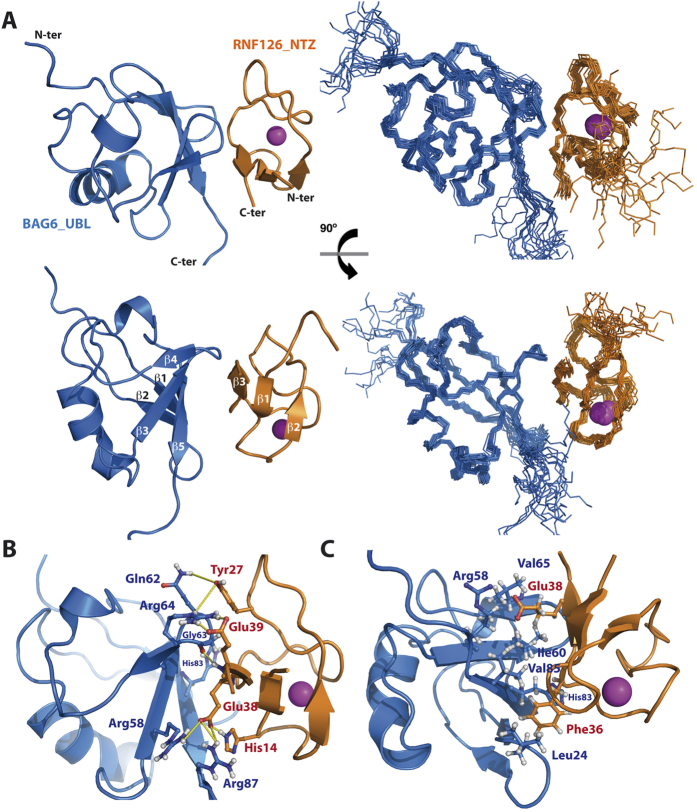
Solution NMR structure of RNF126_NZF/BAG6_UBL complex. (**A**) Orthogonal views of ensemble backbone and cartoon representations showing the 20 lowest energy ARIA-calculated complex structures as deposited in the PDB (Accession number: 2N9P). RNF126_NZF is coloured orange and BAG6_UBL is coloured blue with coordinated zinc cation shown as magenta sphere. (**B**) Network of interactions formed at the complex interface. Residues shown in ball and stick representation are involved in the formation of hydrogen bonds or electrostatic interactions (shown as yellow dashed lines). (**C**) Details of the hydrophobic core of the complex interface. Apolar residues and hydrophobic regions of Glu38 and Arg58 side-chains forming hydrophobic contacts at the binding interface are shown in ball and stick-representation.

The stability of the complex and the high quality NMR data facilitated experimental structure solution of the RNF126_NZF/BAG6_UBL complex with no need to resort to *in silico* methods. We acquired complete triple resonance experiments for backbone and side-chain assignment of the bound state for each protein (BMRB Accession Number: 25914) and we used 3D NOESY experiments to calculate the complex structure (PDB Accession Number: 2N9P; statistical parameters in Table 2). We unambiguously assigned reciprocal intermolecular NOEs between RNF126 and BAG6 ensuring the efficacy of the structure calculation. The complex shows overall conservation of the original fold for each protein and a perfect match between the binding interface of the obtained structure with our CSP analysis (Fig. 3A). RMSD values between the free form of RNF126_NZF (this work) and the bound state are low (backbone RMSD = 0.62) and the zinc coordination parameters are conserved (Table S2); BAG6 also presents low values of RMSD comparing free (PDB: 1WX9) and bound states (backbone RMSD = 1.43), but in this case there is a minor modification of loop β_1_-β_2_ configuration (Figure S6). A variety of interactions is observed in the interface between both proteins including hydrogen bond formation and electrostatic and hydrophobic interactions, consistent with our ITC data (Fig. 2C). A complex hydrogen bond network is detected involving the guanidinium groups of three arginine residues in BAG6 and the carboxyl group of two glutamic acids in RNF126: Arg64 side-chain forms hydrogen bonds with Glu39 and Glu38 is similarly stabilised by Arg58 and Arg87; moreover the Glu38 amide is in hydrogen bond formation with the Gly63 backbone carbonyl group from BAG6. Tyr27 from RNF126, which is aligned with Gln62 from BAG6 stabilising its aromatic ring, interacts with Arg64 through the hydroxyl group. Interactions also occur for imidazole groups of His14 (RNF126) and His83 (BAG6) (Fig. 3B). Hydrophobic contacts are detected between the two components: the aliphatic side-chain of Glu38 from β_3_ of RNF126 packs against Ile60, Val65 and side-chain of Arg58 from BAG6, and Phe36 in β_2_ of RNF126 fits neatly within a hydrophobic pocket created by Leu24, His83 and Val85 from BAG6 (Fig. 3C).

### RNF126 and SGTA compete for the same binding site on BAG6

**Figure 4:**
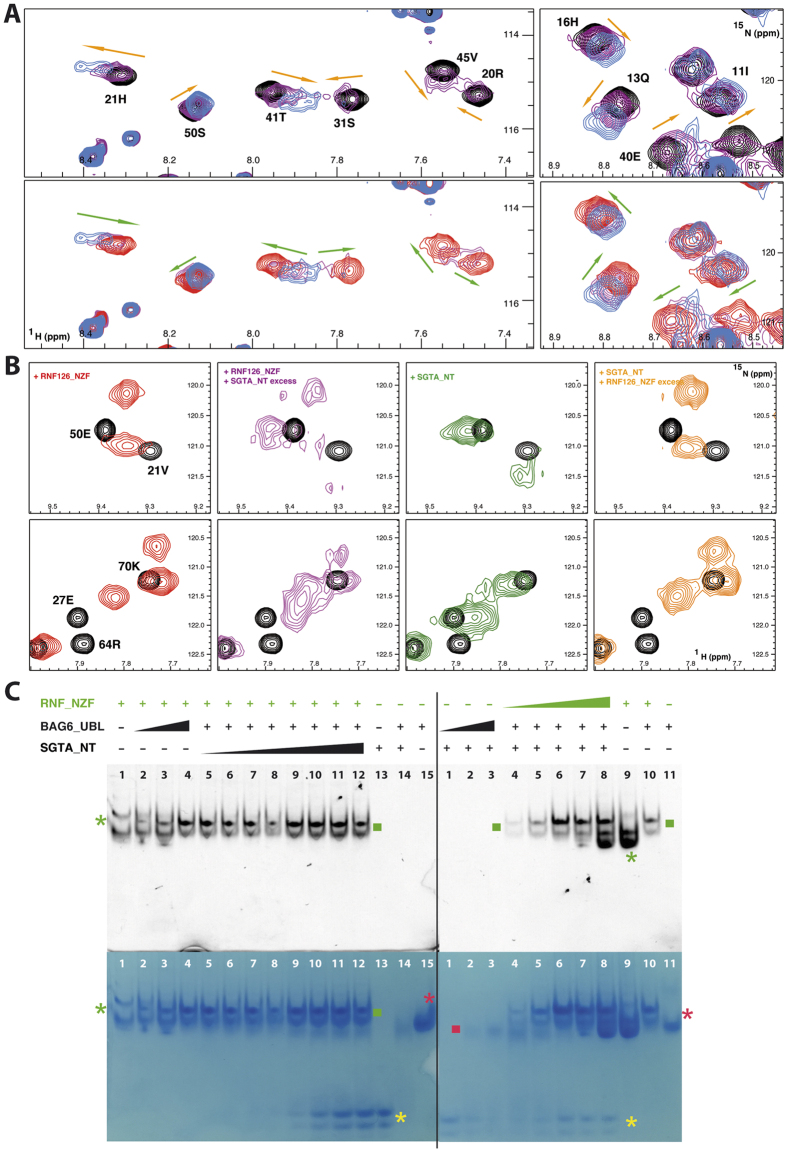
RNF126_NZF competes with SGTA_NT for BAG6_UBL. (**A**) Upper panel - Detailed views of overlapping ^1^H-^15^N HSQC spectra showing ^15^N-labelled SGTA_NT protein titrated with unlabelled BAG6_UBL at ratios: 1:0 (black), 1:1 (purple) and 1:2 (blue). Lower panel – equivalent views of ^1^H-^15^N HSQC spectra of ^15^N-labelled SGTA in complex with unlabelled BAG6_UBL (1:2 molar ratio) titrated with unlabelled RNF126_NZF at ratios: 1:2:0 (blue), 1:1:1 (magenta) and 1:0.5:1.5 (red). (**B**) Detailed views of ^1^H-^15^N HSQC spectra of ^15^N-labelled BAG6_UBL (black) titrated with RNF126_NZF (red, far left) at a ratio of 1:1 and then saturated with unlabelled SGTA_NT (purple) to reach a BAG6:RNF126:SGTA ratio of 1:1:4; or 15-N labelled BAG6_UBL titrated first with unlabelled SGTA_NT (green) at 1:2 ratio and then saturated with unlabelled RNF126_NZF (orange, far right) at ratios of 1:2:2 (BAG6:SGTA:RNF126). Note that since SGTA_NT dimerises, a two-fold concentration of this protein is necessary to reach equivalent concentrations for BAG6/SGTA complex formation. (**C**) Native PAGE of GFP-tagged RNF126_NZF competitive binding assay with BAG6_UBL and SGTA_NT visualised by fluorescence (top) and Coomassie (bottom). Left panel: lane 1 - free GFP-tagged RNF126_NZF, lanes 2–4 - GFP-tagged RNF126_NZF titrated with BAG6_UBL at 0.2, 0.3 and 1 molar equivalents, lanes 5–12 – GFP-tagged RNF126_NZF in complex with BAG6_UBL (1:1 ratio) titrated with SGTA_NT dimer at 0.1, 0.2, 0.3, 0.5, 1, 2, 3 and 4 molar equivalents, lane 13 – free SGTA_NT, lane 14 – SGTA_NT in complex with BAG6_UBL (1:1) and lane 15 – free BAG6_UBL. Right panel: lane 1 – free SGTA, lanes 2 and 3 – SGTA_NT titrated with BAG6_UBL at 0.3 and 1 molar equivalents, lanes 4–8 - SGTA_NT in complex with BAG6_UBL (1:1) titrated with GFP-tagged RNF126_NZF at 0.2, 0.5, 1, 3 and 4 molar equivalents, lane 9 - free GFP-tagged RNF126_NZF, lane 10 - GFP-tagged RNF126_NZF in complex with BAG6_UBL (1:1), and lane 11 – free BAG6_UBL. Free GFP-tagged RNF126_NZF (green), free BAG6_UBL (red) and free SGTA_NT (yellow) are marked with asterisks; GFP tagged RNF_126_NZF/BAG6_UBL complex with a green square and SGTA_NT/BAG6_UBL complex with a red square.

Since the RNF126 binding site on BAG6 defined here showed some overlap with its SGTA interaction interface^15^,^31^, we explored the possibility of simultaneous or competitive binding between these three proteins. First direct binding between SGTA and RNF126_NZF was excluded by reciprocal NMR titrations, which showed unperturbed spectra of each protein upon addition of the other, to a molar ratio of 1:4 (SGTA dimer: RNF) (Figure S7 for the ^15^N-labelled SGTA perspective and data not shown for the reciprocal titration). Next we analysed the CSP of ^15^N-labelled SGTA_NT upon titration with BAG6_UBL and subsequent saturation with RNF126_NZF; the peaks that are perturbed upon BAG6_UBL binding incrementally return to their free state upon RNF126_NZF addition (Figs 4A and S8). Finally, ^15^N-labelled BAG6_UBL protein was titrated with SGTA and then saturated with RNF126_NZF with the characteristic shifts for SGTA binding heading towards the RNF126_NZF binding state upon saturation. We also performed reverse saturation by adding RNF126_NZF followed by SGTA, with equivalent results (Fig. 4B) although the BAG6/RNF126 complex signals were never completely lost in this case. We employed native PAGE mobility shift assays to further analyse this competition for BAG6_UBL binding. We saturated SGTA_NT with BAG6_UBL and detected the band shift indicative of complex formation. We added GFP-tagged RNF126 into the protein mixture at increasing concentrations which caused characteristic band shifts for the unbound form of SGTA and subsequent complex formation between RNF126 and BAG6_UBL. Conversely, reverse titration (i.e. BAG6_UBL/RNF126_NZF complex formation followed by SGTA_NT addition) did not show the release of RNF126 from the RNF126/BAG6_UBL complex upon competition with SGTA_NT (Fig. 4C).

These experiments consistently indicate that RNF126_NZF competes with SGTA for the same binding region in BAG6_UBL and eliminate the possibility of RNF126 and SGTA binding to BAG6 simultaneously. In addition, BAG6_UBL binds more tightly to RNF126_NZF than it does to SGTA_NT as the former interaction has K_d_ ≈ 0.4 μM as calculated from ITC whereas the the ITC data for the latter interaction could not be adequately fitted, despite trying numerous experimental conditions, and was only demonstrable by NMR chemical shift perturbation in fast exchange and size exclusion chromatography^31^.

### Interaction of RNF126 with UBL4A_UBL

**Figure 5:**
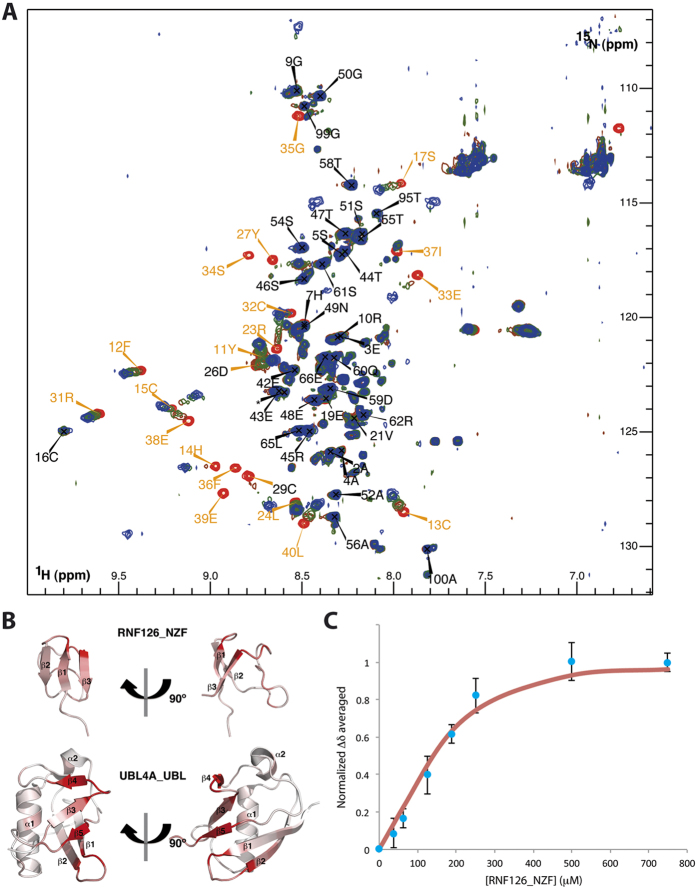
RNF126_NZF interacts with UBL4A_UBL in a manner similar to its interaction with BAG6_UBL, albeit with lower affinity. (**A**) ^1^H-^15^N HSQC spectra of ^15^N-labelled RNF126 1-100 (black) titrated with unlabelled UBL4A_UBL at different molar ratios (RNF126:UBL4A): 1:0 (red), 1:0.125 (brown), 1:0.25 (green), 1:0.75 (light blue) and 1:1 (navy). (**B**) Cartoon views of RNF126_NZF (this paper) and UBL4A_UBL (PDB: 1WX9) coloured according to reciprocal CSP data with the most perturbed residues in red. (**C**) Plot representing the normalized CSP data of the most perturbed UBL4A_UBL amino acids (20, 21, 22, 23, 24, 25, 26, 28, 57, 58, 59, 61, 62, 63, 64, 81, 82, 83, 84, 85, 86) upon titration with different concentrations of RNF126_NZF. Fitting was performed using the DynaFit program^52^ yielding a K_d_ of 16.6 ± 2.7 μM.

Since BAG6_UBL and UBL4A_UBL each bind to SGTA^31^ and are both displayed by the BAG6 complex, we speculated that there might be an interaction between RNF126_NZF and UBL4A_UBL and first investigated this possibility using CSP. Upon titration with unlabelled UBL4A_UBL, ^15^N-labelled RNF_NZF showed chemical shift perturbations within the first forty residues that compared well with those shifts that occur upon BAG6_UBL binding (Fig. 5A). The analysis of the reciprocal titration with ^15^N-labelled UBL4A_UBL was performed using assignments from the BMRB database (Accession Number: 11279). They revealed that the binding interface on UBL4A is equivalent to the one obtained for BAG6: β_3_, β_4_, β_5_ strands and the loop between β_1_ and β_2_ are perturbed upon binding RNF126 (Figs 5B and S3B). Both titrations occupy a fast exchange timescale implying that this interaction has a lower affinity than that of BAG6_UBL binding; in fact, plotting the CSP data against the concentration of RNF126_NZF and fitting the curve to a simple 1:1 equimolar binding model yields a dissociation constant (K_d_) in the order of 20 μM, around 50 times higher than that of BAG6 (Fig. 5C). We also investigated this interaction using ITC but, in the same experimental conditions as were used for BAG6, the observed transition was small and impossible to fit, in keeping with the reduced affinity that we observed (data not shown). MST and native PAGE mobility shift assays also demonstrated a lower affinity or no binding, respectively, between UBL4A and RNF126 as compared to the BAG6 binding (Figures S4).

**Figure 6:**
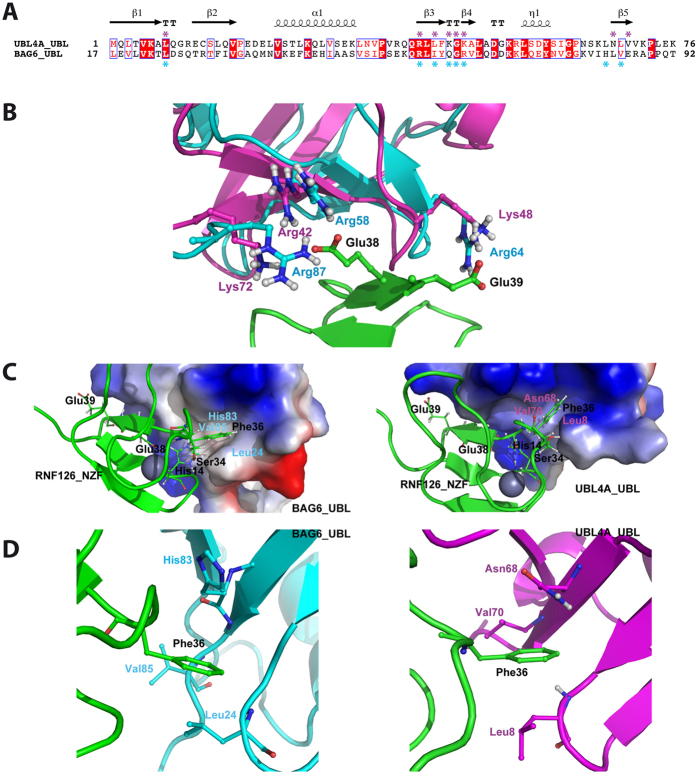
Detailed comparison of BAG6_UBL and UBL4A_UBL binding to RNF126_NZF. (**A**) Structure-based sequence alignment of BAG6_UBL and UBL4A_UBL with the most perturbed residues from the CSP analysis indicated with asterisks; boxes show conserved residues while red highlights sequence identity, structural motifs are labelled across the top with ‘TT’ indicating a β turn. Figure generated using ESPript 3.0 server. (**B**) Superimposition of the BAG6_UBL complex with RNF126_NZF (experimentally calculated using ARIA) with the HADDOCK generated complex of UBL4A_UBL and RNF126_NZF highlighting putative differences in electrostatic interactions at the binding interface. BAG6 is coloured in cyan, UBL4A in magenta and RNF126 in green, with polar residues at the interface shown in ball and stick representation. (**C**) Vacuum electrostatics view of UBL binding pockets (BAG6 on the right and UBL4A on the left) indicating higher hydrophobicity of BAG6_UBL compared to that of UBL4A_UBL. RNF126 is shown in cartoon representation (green) with the zinc cation shown as a sphere (blue); RNF126 residues involved in interactions are shown as sticks. (**D**) Detailed view of RNF126_NZF/UBL hydrophobic interactions showing key differences between residues lining the hydrophobic pocket. The backbone NH group of Leu24 and the aliphatic part of the His83 sidechain that lines the cavity in BAG6_UBL suggests stronger hydrophobic interactions with Phe36 in RNF126_NZF (right panel). In contrast, the sidechain NH group of Asn68 lines the cavity in UBL4A_UBL and the backbone amino group of Leu8 is solvent exposed (left panel).

Finally, we generated a structural model of the RNF126_NZF/UBL4A_UBL complex using our CSP data in HADDOCK-based semi-rigid, data-driven docking^32^,^33^. We analysed the three lowest energy HADDOCK clusters of RNF126_NZF/UBL4A_UBL (Figure S9A and Table S3). The top-ranked cluster is highly similar to our experimentally-derived RNF126_NZF/BAG6_UBL structure (Figure S9B) showing that residues making contacts with RNF126_NZF are conserved between UBL4A_UBL and BAG6_UBL (Figs 6A, S9C and S9D). The UBL4A_UBL residues displayed in the binding interface with RNF126 are Leu8, Arg42, Leu44, Lys48, Ala49, Asn68 and Val70 of UBL4A (Figure S9C), which align with residues Leu24, Arg58, Ile60, Arg64, Val65, His83 and Val85 of BAG6_UBL (Figure S9D). Both of these sets of UBL residues make contact with His14, Tyr27, Phe36, Glu38 and Glu39 in RNF126_NZF. The positively charged Lys48, Arg42 and Lys72 of UBL4A (equivalent to Arg64, Arg58 and Arg87 in BAG6) form electrostatic interactions with Glu39 and Glu38 in RNF126_NZF (Fig. 6B). Phe36 of RNF126_NZF inserts into a pocket formed by Leu8, Asn68 and Val70 of UBL4A making hydrophobic contacts between aliphatic portions of the side-chains (Fig. 6C,D). We conclude that, like SGTA, RNF126 is capable of binding to both of the UBL domain containing subunits of the BAG6 complex.

## Discussion

There are hundreds of E3 ubiquitin ligases in mammalian cells to process the wide variety of protein degrons^34^,^35^, but the way in which E3 ubiquitin ligases select their specific substrates remains elusive. RNF126 belongs to the large RING family of ligases, whose defining feature is a zinc-binding RING finger domain which performs the ubiquitin ligase activity through interaction with an E2 (ubiquitin-conjugating) enzyme. RNF126 has been specifically connected with the BAG6 sortase, ubiquitinating hydrophobic substrates to send them down the route towards proteasomal degradation^4^. Many of the RING E3 ligases contain additional zinc-finger motifs which can act as recognition sites for substrates or substrate-bearing delivery systems such as the BAG6 complex. Here we define and characterise a molecular-level interaction between RNF126 and BAG6 and report a further, lower-affinity RNF126 interaction with UBL4A, another subunit of the BAG6 sortase complex.

The structure of the complete heterotrimeric BAG6 complex has yet to be solved and its large size, dynamic behaviour and transient interactions present substantial challenges to the structural biologist. Structures of several isolated domains from different species^31^,^36^,^37^ and partial complexes^19^,^38^,^39^ have shed some light on its function at a molecular level. Nevertheless, much of BAG6 itself is completely unsolved and there are still many unknowns. In this work we determine that RNF126, although capable of binding to two different UBL domains on the BAG6 complex, has a clear preference for the BAG6_UBL.

By comparing the RNF126 binding sites on the BAG6 and UBL4A subunits at a structural level we can gain some insight into the likely basis for these differential binding affinities (Fig. 6C,D) with the caveat that we are comparing a solved NMR complex structure (BAG6) to a solid HADDOCK model derived from solved component structures and chemical shift perturbation data (UBL4A). Notably, the conformation of the loop between the β1 and β2 strands of UBL4A_UBL differs from that of the BAG6_UBL such that its Leu8 sidechain points away from RNF126_NZF Phe36 in the binding cavity. Furthermore the backbone NH group of the UBL4A Leu8 is solvent-exposed and lines this pocket. In contrast, the corresponding backbone NH group of Leu24 in the BAG6_UBL is buried in the hydrophobic core. The sidechain NH group of Asn68 in the UBL4A_UBL also lines this binding cavity whereas it is the aliphatic part of the His83 sidechain that occupies the equivalent space in the BAG6_UBL (Fig. 6C,D). Taken together, these structural differences reduce the hydrophobicity of the RNF126 Phe36 binding cavity in the UBL4A_UBL relative to the BAG6_UBL, which likely decreases the affinity of the former interaction. A further reduction may be caused by the weaker interaction of Lys48 in UBL4A_UBL with the RNF126_NZF Glu38 compared with Arg64 of BAG6_UBL which has the space to form more hydrogen bonds (Fig. 6B).

**Figure 7:**
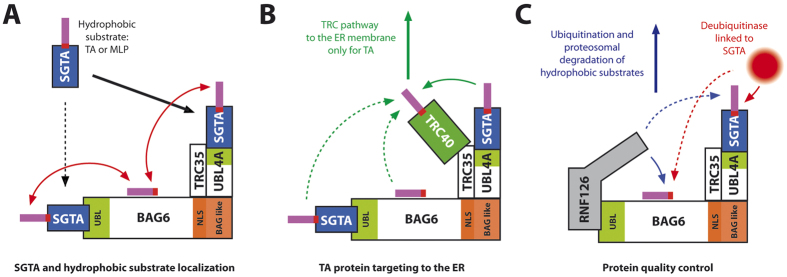
Schematic illustration of quality control pathways for hydrophobic proteins in the cytoplasm. (**A**) SGTA bearing a hydrophobic substrate reaches the BAG6 complex, likely binding the UBL4A_UBL domain, but possibly the BAG6_UBL. The substrate (TA proteins and/or MLPs) could remain bound to SGTA (and passed directly to heat-shock chaperones that bind the TPR) or be transferred to BAG6. (**B**) TA substrates are transferred to TRC40 for ER membrane insertion likely through SGTA bound to UBL4A which binds TRC35 and recruits TRC40. (**C**) At the same time, BAG6 is involved in protein quality control of hydrophobic substrates. RNF126_NZF binds BAG6_UBL, possibly replacing SGTA and, in concert with E2 enzymes could ubiquitinate the hydrophobic substrates from BAG6 or SGTA, leading them to proteasomal degradation. This process is regulated by the opposite process of deubiquitination in which SGTA may have a recruiting role at least at the proteasome level.

Earlier studies^14^,^31^ identified a comparable situation to the one described here for RNF126, in which the cochaperone SGTA can also bind to the UBLs of both BAG6 and UBL4A, in the same region as RNF126 (Figure S10), but, in this case, with the opposite preference i.e. more tightly with UBL4A than BAG6. The UBL binding site on SGTA looks significantly different to that of RNF126 with the UBL-binding capability of SGTA seemingly more dependent on electrostatic interactions, which are stronger for UBL4A_UBL^29^,^31^, providing a molecular basis for its relative preference. This delicate balance of binding affinities might be key in determining the dynamic process of decision-making at the BAG6 complex (Fig. 7).

In contrast to the analogous Get4/Get5 complex in yeast which displays one type of UBL (located at the centre of each Get5 subunit), the mammalian BAG6 complex has two types of UBL to which SGTA can deliver hydrophobic substrates, and it now appears that these may be functionally distinct^19^,^38^. In particular, it seems that only a small C-terminal portion of the BAG6 protein that brings together the UBL4A and TRC35 subunits is required to enable the transfer of TA proteins from SGTA to the TRC40 delivery complex^19^. Given that SGTA is the first port of call for newly synthesised TA proteins^36^, and SGTA seems to preferentially bind to the UBL4A_UBL subunit of the BAG6 complex, how are hydrophobic polypeptide clients that are destined for quality control passed on to the BAG6 subunit in order to enable their RNF126 mediated ubiquitination? Both SGTA and the BAG6 complex can interact with the same mislocalised membrane protein (MLP) model^16^, confirming the possibility of substrate transfer between them (Fig. 7). Therefore, based on the analogy of TA proteins, one possibility is that SGTA can present quality control clients to the BAG6 complex via the UBL4A_UBL, and it is an inability of TRC40 to collect these substrates that enables their redirection to the BAG6 subunit^40^. In this case, the well-defined TMD of TA proteins would ensure that they are quickly passed along from the C-terminal domain of SGTA via a UBL4A mediated interaction with the BAG6 complex to TRC40 and hence taken onwards for ER delivery. The failure of substrates such as MLPs to be efficiently collected from SGTA by TRC40 would instead direct them towards the BAG6 subunit and thereby elicit entry into the alternative quality control pathway (Fig. 7).

Alternatively, it is possible that SGTA can “sense” the nature of the bound client, for example, by detecting unfolded or non-native hydrophobic regions. In the case of aberrant clients such as MLPs, such recognition might increase the preference of SGTA for the BAG6_UBL, thereby ensuring the direct transfer of these substrates to the BAG6 subunit for quality control. The BAG6 subunit is certainly capable of substrate discrimination as moderately hydrophobic polypeptides are known to bind close to its N-terminal UBL domain^41^ while membrane proteins and heat denatured luciferase bind its central proline rich region^15^,^42^. In the case of MLPs, we predict that competition between SGTA and RNF126 for binding to the BAG6_UBL influences the stability of these substrates since any increase in quantity or residence time of SGTA would reduce the access of RNF126 to its BAG6-bound clients, thereby reducing their ubiquitination and inhibiting their proteasomal degradation. This model is consistent with both the reduction in BAG6 client polyubiquitination and delay in MLP degradation that are observed upon overexpression of wild type SGTA but not of an SGTA mutant that is defective in binding to the BAG6 complex^16^.

The importance of the N-terminal UBL of BAG6 in recruiting quality control factors is underlined by the observation that it is also wholly (in the case of Hrd1) or partly (in the case of Gp78) responsible for its interaction with two, membrane-bound E3 ligases, which are involved in ERAD^43^. We were therefore struck by the fact that another soluble E3 ligase RNF115 shares near identical NZF and RING domains with RNF126 (with 42.3% overall sequence identity and 52.6% similarity) (Figure S11). We speculate that RNF115 is highly likely to bind to the BAG6_UBL, perhaps conferring some functional redundancy on its role in the selective ubiquitination of MLPs as suggested by the incomplete effects that are observed following RNF126 depletion both *in vitro* and *in vivo*^4^.

This study therefore contributes towards our understanding of the way in which hydrophobic proteins are sorted between distinct cytosolic quality control pathways. Further work will help us fathom the process of selection between all the possible interactions that can occur at the BAG6 complex. Furthermore, the client specificity of E3 ligases is proposed to limit the potential side-effects of drugs that target them, and the new RNF126 structure presented here provides a potential starting point for developing a targeted anti-cancer therapy^25^,^44^,^45^.

## Methods

### Plasmid preparation

Gene fragments encoding human RNF126 (1–40 and 1–100) and N-terminal SGTA (1–86) were PCR amplified from cDNA (Life Technologies) and cloned into *Bam*HI/*Xho*I restriction sites of a home-modified pET28 vector which encodes an N-terminal thioredoxin A fusion protein followed by a hexahistidine tag and tobacco etch virus (TEV) protease cleavage site. GFP versions of RNF124 (1–40) were cloned following a similar strategy but using a pET28 vector variant fused with N-terminal GFP instead of thioredoxin A. The RNF126 mutants (H14A, F36A, E38K and E39K) were obtained by PCR mutagenesis reaction using pET28-GFP-RNF126 (1–40) vector as the template and different oligonucleotides carrying the mutated codons. Sequences corresponding to the UBL domains of BAG6 (17–101) and UBL4A (1–74) were amplified and inserted via ligation-independent Ek/LIC cloning (Novagen) into a pET46 vector.

### Protein production

SGTA_NT and UBL4A_UBL plasmids were transformed into *E. coli* Rosetta cells, while BAG6_UBL and RNF126 ones were transformed into BL21 (DE3) strains. Typically, protein expression was induced by adding 0.3–0.5 mM isopropyl-β-D-thiogalactopyranoside (IPTG) to bacterial cultures at OD_600_ ≈ 0.8, followed by either 4 hours incubation at 37 °C (SGTA, UBL4A and BAG6) or overnight at 18 °C (RNF126). For labelled proteins, growth was carried out in M9 media supplemented with labelled ammonium chloride (>98% ^15^N, Sigma-Aldrich) and/or glucose (>99% U-^13^C, Sigma-Aldrich). For RNF126 expression, the cultures were supplemented with 10 μM ZnCl_2_.

Harvested cells were resuspended in lysis buffer (20 mM potassium phosphate, pH 8.0, 300 mM NaCl, 10 mM Imidazole, 250 μM TCEP), supplemented with protease inhibitors (0.3 μM Aprotinin, 10 μM Leupeptin and 1 μM Pepstatin A) and 1mM PMSF, and lysed by sonication or using a cell disruptor (Constant Systems Ltd). Cell debris and insoluble material were removed by centrifugation and overexpressed protein recovered from soluble fractions was purified using nickel affinity chromatography (HisTrap^TM^ HP 5 ml, GE Healthcare). Recombinant proteins were eluted with buffer containing 300 mM imidazole, then dialyzed against cleavage buffer (20 mM potassium phosphate, pH 8.0 and 300 mM NaCl) and simultaneously digested with homemade TEV protease (≈100 μg/ml) at 4 °C overnight. After TEV cleavage a second nickel affinity chromatography was performed to remove fusion protein, histidine tags, undigested protein and TEV protease; the desired protein was then recovered in the flow through and loaded into a HiLoad 16/60 Superdex 75 column (GE Healthcare), previously equilibrated in buffer containing 10 mM potassium phosphate pH 6.0, 100 mM NaCl and 250 μM TCEP, for a final gel filtration step. Proteins were concentrated using Vivaspin concentrators (Sartorius Stedin) and sample purity and homogeneity was checked by SDS-PAGE, mass spectrometry and NMR.

### NMR structural experiments

Protein samples at concentrations between 100 and 1000 μM were prepared in 10% or 100% D_2_O (Sigma Aldrich), 10 mM potassium phosphate pH 6.0, 100 mM NaCl and 250 μM TCEP buffer (also containing 10 μM DSS for proton chemical shift referencing). All NMR experiments were acquired in 5 mm NMR tubes at 25 °C on Bruker Avance spectrometers operating at 500 MHz and 700 MHz equipped with cryoprobes, controlled by the TopSpin 3.1 software package. Backbone assignments were carried out using 3D experiments (HNCO, HN(CA)CO, CBCA(CO)NH, and CBCANH)^46^ and side-chain resonances were assigned using 3D HCCH-TOCSY experiments for both RNF126_NZF and for the complex of RNF126_NZF together with BAG6_UBL. All NMR spectra were processed with NMRPipe^47^ and analysed with CcpNMR Analysis^48^.

NOE distance restraints were derived from different NOESY experiments: 2D NOESY (in 90% H_2_O and 100% D_2_O) for RNF126_NZF; and two pairs of ^15^N-edited NOESY-HSQC, ^13^C-edited NOESY-HSQC and ^12^C, ^14^N-filtered-^13^C-edited NOESY-HSQC experiments for the complex using either ^15^N, ^13^C labelled BAG6_UBL or RNF126 1–40. Dihedral constraints (ϕ and Ψ angles) were extracted from the chemical shift values using TALOS+ program^49^. The structure calculation was performed using the ARIA2 program^50^ by generating 100 conformers in the final iteration and selecting the 20 best structures with lowest restraint energies for a final step of refinement in water. The coordination sphere of the zinc atom in RNF126 structure was obtained in a first round of calculations using only NOE based distance restraints and once it was confirmed, the zinc atom coordination was incorporated as an additional restraint in calculations that followed using ARIA2 tools. The final ensemble of each structure (PDB: 2N9O; RNF126_NZF, 2N9P; RNF_NZF/BAG6_UBL) was analysed and represented using MOLMOL^51^ and PyMOL (DeLano Scientific LLC, Palo Alto, CA, USA).

### NMR titrations

Proteins used for NMR titrations (RNF126 constructs, UBL4A_UBL, BAG6_UBL and SGTA) were dialysed overnight in the same buffer (10 mM potassium phosphate pH 6.0, 100 mM NaCl and 250 μM TCEP) and mixed in different molar ratios keeping the concentration of the labelled protein constant. Typically, ^1^H-^15^N HSQC experiments were recorded for each titration point at 25 °C and CSP calculated for each amide signal using the following formula, where Δδ_1H_ and Δδ_15N_ are the chemical shift differences for the same amide in its free and bound spectra (δ_free_-δ_bound_) and for proton and nitrogen values respectively:


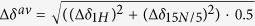


CSP results were mapped onto the structures using the PyMOL software. For the RNF126 titration with UBL4A_UBL, chemical shift perturbation data were analysed and fitted using the Dynafit software^52^.

### ITC

ITC experiments were performed at 25 °C using an ITC-200 MicroCal microcalorimeter (GE Healthcare) following the standard procedure as reported previously^31^. Proteins were prepared in 10 mM potassium phosphate pH 6.0, 100 mM NaCl, 250 μM TCEP. In each titration, 20 injections of 2 μL of RNF126_NZF (wild-type or mutant), each at a concentration of 500 μM, were added to a sample of BAG6_UBL or UBL4A_UBL at 50 μM in the reaction cell. Integrated heat data obtained for the titrations, corrected for heats of dilution, were fitted using a nonlinear least-squares minimization algorithm to a theoretical titration curve, using the MicroCal-Origin 7.0 software package. ΔH (reaction enthalpy change in Kcal/mol), K_b_ (equilibrium binding constant per molar), and n (molar ratio between the proteins in the complex) were the fitting parameters. The reaction entropy, ΔS, was calculated using the relationships ΔG = −RT⋅lnK_b_ (*R* = 8.314 J/(mol⋅K), T 298 K) and ΔG = ΔH−TΔS. Dissociation constants (*K*_d_) are shown for each interaction.

### MST

Microscale thermophoresis protein-protein interaction studies were performed on the Monolith NT.115 (Nanotemper Technologies, Munich, Germany) using GFP tagged version of RNF126_NZF under these buffer conditions: 10 mM potassium phosphate, pH 6.0, 100 mM NaCl, 250 μM TCEP. Samples were prepared mixing GFP tagged RNF126_NZF with either BAG6_UBL or UBL4A_UBL, keeping RNF126 at constant concentrations of 0.125 or 0.250 μM, while UBL domain concentrations ranged from 25 μM to 0.8 nM (BAG6_UBL), or 50 μM to 1.5 nM (UBL4A_UBL) using 1:1 serial dilutions. After a 15 minute incubation, ~5 μl of each solution was loaded into Monolith NT Standard Capillaries (NanoTemper Technologies GmbH). Thermophoresis rates were measured at an ambient temperature of 25 °C with 5s/30s/5s laser off/on/off times, respectively. Instrument parameters were adjusted with 10–20% LED power and 20% IR-laser power. Data from three independently pipetted measurements were analyzed (NT Analysis software version 1.2.101, NanoTemper Technologies) using the signal from Thermophoresis + T-Jump.

### Native PAGE mobility shift assay

Native PAGE mobility shift assay was used in the analysis of protein-protein interactions. The experiment was performed by incubating 25 μM GFP tagged RNF126_NZF (wild type and mutant variants) with increasing amounts of BAG6_UBL or UBL4A_UBL (at a concentration range between 0 to 4 molar equivalents) for 10 min at room temperature in a final volume of 20 μl. In the case of competition experiments, GFP tagged RNF126 was added to the SGTA_NT and BAG6_UBL protein mixture at increasing concentrations (between 0 to 4 molar equivalents). In reverse titrations, increasing amounts of SGTA_NT were added to the RNF126/BAG6_UBL complex. Native gels were prepared using 10% polyacrylamide in 0.45 M Tris-HCl pH 8.8, 1% ammonium persulphate and 0.02% TEMED and run under native conditions, in buffer containing 25 mM Tris, 200mM glycine at 100 V for 180 min. Fluorescent bands were visualized using an Amersham Imager 600 (GE Healthcare) with a light source of Epi-RGB green light at 520 nm and Cy3 emission filter. The gels were later stained using Coomassie to visualise all protein bands.

### RNF126_NZF/UBL4A complex assembly using HADDOCK

Chemical shift perturbation studies defined clear interaction surface areas in the RNF126_NZF/UBL4A_UBL complex. This data was used for complex structure calculation using the HADDOCK approach. For the calculation, PDB-deposited structures of UBL4A_UBL (2DZI) and the lowest energy NMR structure from our family of RNF126_NZF structures were used. Ambiguous Interaction Restraints (AIRs) were implemented according to the standard protocol. The chemical shift perturbation data enabled identification of 14 amino acid residues in RNF126_NZF and 21 in UBL4A_UBL with chemical shift changes greater than 0.15 ppm. After filtering for a relative solvent accessibility higher than 45%, calculated using the program Naccess, 10 residues in RNF126_NZF and 14 in UBL4A respectively, were identified as active. These were RNF126_NZF residues 14, 27, 34, 36, 38, 39 and UBL4A residues 8, 44, 48, 49, 68, 70. Solvent exposed residues juxtaposed to the active residues were automatically termed passive residues by the HADDOCK protocol. One thousand initial complex structures were generated by rigid body energy minimization, and the best 200 (lowest total energy) were selected for torsion angle dynamics and subsequent Cartesian dynamics in an explicit water solvent. Default scaling for energy terms was applied. Following the standard benchmarked protocol, cluster analysis yielded 175 structures in 5 cluster ensembles. The top scoring cluster (lowest energy) was taken as the most reliable result as shown by HADDOCK benchmark testing.

## Additional Information

**How to cite this article**: Krysztofinska, E. M. *et al.* Structural and functional insights into the E3 ligase, RNF126. *Sci. Rep.* **6**, 26433; doi: 10.1038/srep26433 (2016).

## Supplementary Material

Supplementary Information

## Figures and Tables

**Table 1:** NMR and refinement statistics for the final 20 structure ensembles of RNF126_NZF.

**NMR distance and dihedral restraints**		
*Distance restraints*		
Total NOE	719	
Intra-residue	326	
Sequential (|i-j| = 1)	119	
Medium-range (1 < |i-j| < 4)	55	
Long-range (|i-j| > 5)	219	
*TALOS derived dihedral restraints*		
Total dihedral restraints (Φ+Ψ)	34	
Structure statistics		
*Violations (mean and s.d.)*		
Number of violated distance restraints per structure (>0.15 Å)	1.2 ± 0.9	
Max. distance restraint violation (Å)	0.19	
Number of violated dihedral angle restraints per structure (>2°)	5.1 ± 0.6	
Max. dihedral angle restraint violation (°)	2.8	
Ramachandran Plot analysis (%)*	*(1–40)*	*(10–40)*
Residues in most favoured regions	77.4	84.8
Residues in additionally allowed regions	19.8	15.2
Residues in generously allowed regions	0.9	0.0
Residues in disallowed regions	1.8	0.0
*Derivation from idealized geometry*		
Bond length (Å)	0.0041 ± 0.0001	
Bond angles (°)	0.58 ± 0.01	
*Averages RMSD to mean structure (range)*	*(10–40)*	
Backbone (Å)	0.19 ± 0.05	
Heavy (Å)	0.73 ± 0.11	

*Obtained from PROCHECK-NMR.

**Table 2:** NMR and refinement statistics for the final 20 structure ensembles of RNF126_NZF/BAG6_UBL complex.

**NMR distance and dihedral constrains**		
*Intramolecular distance constrains*	RNF126_NZF	BAG6_UBL
Total unambiguous NOEs	461	1283
Intra-residue	252	689
Sequential (|i-j| = 1)	58	266
Medium-range (1 < |i-j| < 4)	23	115
Long-range (|i-j| > 5)	128	213
Ambiguous constrains	2	51
*Intermolecular distance constrains*	48	
*TALOS derived dihedral constrains*	RNF126_NZF	BAG6_UBL
Total dihedral constrains (Φ+Ψ)	33	90
Structure statistics		
*Violations (mean and s.d.)*		
Number of violated distance restraints per structure (>0.25 Å)	1.2 ± 0.5	
Max. distance constraint violation (Å)	0.28	
Number of violated dihedral angle restraints per structure (>5°)	1.3 ± 0.8	
Max. dihedral angle violation (°)	7.6	
Ramachandran Plot analysis (%)*	*RNF126 (1–40) BAG6 (1–101)*	*RNF126 (10–40) BAG6 (17–88)*
Residues in most favoured regions	65.3	72.3
Residues in additionally allowed regions	28.6	22.5
Residues in generously allowed regions	3.9	3.8
Residues in disallowed regions	2.2	1.3
*Derivation from idealized geometry*		
Bond length (Å)	0.0086 ± 0.0003	
Bond angles (°)	0.84 ± 0.02	
*Averages RMSD to mean structure (range)*	*RNF126 (10–40)/BAG6 (17–88)*	
Backbone (Å)	0.97 ± 0.25	
Heavy (Å)	1.64 ± 0.22	

*Obtained from PROCHECK-NMR.

## References

[b1] Takalo M., Salminen A., Soininen H., Hiltunen M. & Haapasalo A. Protein aggregation and degradation mechanisms in neurodegenerative diseases. Am J Neurodegener Dis 2, 1–14 (2013). 23516262 PMC3601466

[b2] Shao S. & Hegde R. S. Membrane protein insertion at the endoplasmic reticulum. Annu Rev Cell Dev Biol 27, 25–56, 10.1146/annurev-cellbio-092910-154125 (2011). 21801011 PMC4163802

[b3] Johnson N., Powis K. & High S. Post-translational translocation into the endoplasmic reticulum. Biochim Biophys Acta 1833, 2403–2409, 10.1016/j.bbamcr.2012.12.008 (2013). 23266354

[b4] Rodrigo-Brenni M. C., Gutierrez E. & Hegde R. S. Cytosolic quality control of mislocalized proteins requires RNF126 recruitment to Bag6. Molecular cell 55, 227–237, 10.1016/j.molcel.2014.05.025 (2014). 24981174 PMC4104027

[b5] Kriegenburg F., Poulsen E. G., Koch A., Kruger E. & Hartmann-Petersen R. Redox control of the ubiquitin-proteasome system: from molecular mechanisms to functional significance. Antioxid Redox Signal 15, 2265–2299, 10.1089/ars.2010.3590 (2011). 21314436

[b6] Valastyan J. S. & Lindquist S. Mechanisms of protein-folding diseases at a glance. Dis Model Mech 7, 9–14, 10.1242/dmm.013474 (2014). 24396149 PMC3882043

[b7] McClellan A. J., Scott M. D. & Frydman J. Folding and quality control of the VHL tumor suppressor proceed through distinct chaperone pathways. Cell 121, 739–748, 10.1016/j.cell.2005.03.024 (2005). 15935760

[b8] Murata S., Minami Y., Minami M., Chiba T. & Tanaka K. CHIP is a chaperone-dependent E3 ligase that ubiquitylates unfolded protein. EMBO Rep 2, 1133–1138, 10.1093/embo-reports/kve246 (2001). 11743028 PMC1084164

[b9] Hartl F. U. Chaperone-assisted protein folding: the path to discovery from a personal perspective. Nat Med 17, 1206–1210, 10.1038/nm.2467 (2011). 21989011

[b10] Tanaka E., Nemoto T. K. & Ono T. Liberation of the intramolecular interaction as the mechanism of heat-induced activation of HSP90 molecular chaperone. Eur J Biochem 268, 5270–5277 (2001). 11606188 10.1046/j.0014-2956.2001.02458.x

[b11] Mariappan M. *et al.* A ribosome-associating factor chaperones tail-anchored membrane proteins. Nature 466, 1120–1124, 10.1038/nature09296 (2010). 20676083 PMC2928861

[b12] Borgese N. & Fasana E. Targeting pathways of C-tail-anchored proteins. Biochim Biophys Acta 1808, 937–946, 10.1016/j.bbamem.2010.07.010 (2011). 20646998

[b13] Hessa T. *et al.* Protein targeting and degradation are coupled for elimination of mislocalized proteins. Nature 475, 394–397, 10.1038/nature10181 (2011). 21743475 PMC3150218

[b14] Xu Y., Cai M., Yang Y., Huang L. & Ye Y. SGTA recognizes a noncanonical ubiquitin-like domain in the Bag6-Ubl4A-Trc35 complex to promote endoplasmic reticulum-associated degradation. Cell reports 2, 1633–1644, 10.1016/j.celrep.2012.11.010 (2012). 23246001 PMC3534891

[b15] Leznicki P. *et al.* The association of BAG6 with SGTA and tail-anchored proteins. PloS one 8, e59590, 10.1371/journal.pone.0059590 (2013). 23533635 PMC3606182

[b16] Wunderley L., Leznicki P., Payapilly A. & High S. SGTA regulates the cytosolic quality control of hydrophobic substrates. Journal of cell science 127, 4728–4739, 10.1242/jcs.155648 (2014). 25179605 PMC4215715

[b17] Leznicki P. & High S. SGTA antagonizes BAG6-mediated protein triage. Proceedings of the National Academy of Sciences of the United States of America 109, 19214–19219, 10.1073/pnas.1209997109 (2012). 23129660 PMC3511132

[b18] Leznicki P., Clancy A., Schwappach B. & High S. Bat3 promotes the membrane integration of tail-anchored proteins. Journal of cell science 123, 2170–2178, 10.1242/jcs.066738 (2010). 20516149 PMC2886740

[b19] Mock J. Y. *et al.* Bag6 complex contains a minimal tail-anchor-targeting module and a mock BAG domain. Proceedings of the National Academy of Sciences of the United States of America 112, 106–111, 10.1073/pnas.1402745112 (2015). 25535373 PMC4291651

[b20] Mateja A. *et al.* Protein targeting. Structure of the Get3 targeting factor in complex with its membrane protein cargo. Science 347, 1152–1155, 10.1126/science.1261671 (2015). 25745174 PMC4413028

[b21] Vilardi F., Stephan M., Clancy A., Janshoff A. & Schwappach B. WRB and CAML are necessary and sufficient to mediate tail-anchored protein targeting to the ER membrane. PloS one 9, e85033, 10.1371/journal.pone.0085033 (2014). 24392163 PMC3879356

[b22] Yamamoto Y. & Sakisaka T. Molecular machinery for insertion of tail-anchored membrane proteins into the endoplasmic reticulum membrane in mammalian cells. Molecular cell 48, 387–397, 10.1016/j.molcel.2012.08.028 (2012). 23041287

[b23] Wang Q. *et al.* A ubiquitin ligase-associated chaperone holdase maintains polypeptides in soluble states for proteasome degradation. Molecular cell 42, 758–770, 10.1016/j.molcel.2011.05.010 (2011). 21636303 PMC3138499

[b24] Claessen J. H. & Ploegh H. L. BAT3 guides misfolded glycoproteins out of the endoplasmic reticulum. PloS one 6, e28542, 10.1371/journal.pone.0028542 (2011). 22174835 PMC3234288

[b25] Zhi X. *et al.* E3 ubiquitin ligase RNF126 promotes cancer cell proliferation by targeting the tumor suppressor p21 for ubiquitin-mediated degradation. Cancer Res 73, 385–394, 10.1158/0008-5472.CAN-12-0562 (2013). 23026136

[b26] Smith C. J., Berry D. M. & McGlade C. J. The E3 ubiquitin ligases RNF126 and Rabring7 regulate endosomal sorting of the epidermal growth factor receptor. Journal of cell science 126, 1366–1380, 10.1242/jcs.116129 (2013). 23418353

[b27] Smith C. J. & McGlade C. J. The ubiquitin ligase RNF126 regulates the retrograde sorting of the cation-independent mannose 6-phosphate receptor. Experimental cell research 320, 219–232, 10.1016/j.yexcr.2013.11.013 (2014). 24275455

[b28] Simon A. C. *et al.* 1H, 13C and 15N assignments of Sgt2 N-terminal dimerisation domain and its binding partner, Get5 Ubiquitin-like domain. Biomolecular NMR assignments 7, 271–274, 10.1007/s12104-012-9425-7 (2013). 23001946

[b29] Simon A. C. *et al.* Structure of the Sgt2/Get5 complex provides insights into GET-mediated targeting of tail-anchored membrane proteins. Proceedings of the National Academy of Sciences of the United States of America 110, 1327–1332, 10.1073/pnas.1207518110 (2013). 23297211 PMC3557055

[b30] Kornhaber G. J., Snyder D., Moseley H. N. & Montelione G. T. Identification of zinc-ligated cysteine residues based on 13Calpha and 13Cbeta chemical shift data. Journal of biomolecular NMR 34, 259–269, 10.1007/s10858-006-0027-5 (2006). 16645816

[b31] Darby J. F. *et al.* Solution structure of the SGTA dimerisation domain and investigation of its interactions with the ubiquitin-like domains of BAG6 and UBL4A. PloS one 9, e113281, 10.1371/journal.pone.0113281 (2014). 25415308 PMC4240585

[b32] Dominguez C., Boelens R. & Bonvin A. M. HADDOCK: a protein-protein docking approach based on biochemical or biophysical information. Journal of the American Chemical Society 125, 1731–1737, 10.1021/ja026939x (2003). 12580598

[b33] de Vries S. J., van Dijk M. & Bonvin A. M. The HADDOCK web server for data-driven biomolecular docking. Nature protocols 5, 883–897, 10.1038/nprot.2010.32 (2010). 20431534

[b34] Ardley H. C. & Robinson P. A. E3 ubiquitin ligases. Essays Biochem 41, 15–30, 10.1042/EB0410015 (2005). 16250895

[b35] Varshavsky A. Regulated protein degradation. Trends in biochemical sciences 30, 283–286, 10.1016/j.tibs.2005.04.005 (2005). 15950869

[b36] Chartron J. W., VanderVelde D. G. & Clemons W. M. Jr. Structures of the Sgt2/SGTA dimerization domain with the Get5/UBL4A UBL domain reveal an interaction that forms a conserved dynamic interface. Cell reports 2, 1620–1632, 10.1016/j.celrep.2012.10.010 (2012). 23142665 PMC3654831

[b37] Chartron J. W., Gonzalez G. M. & Clemons W. M. Jr. A structural model of the Sgt2 protein and its interactions with chaperones and the Get4/Get5 complex. The Journal of biological chemistry 286, 34325–34334, 10.1074/jbc.M111.277798 (2011). 21832041 PMC3190793

[b38] Kuwabara N. *et al.* Structure of a BAG6 (Bcl-2-associated athanogene 6)-Ubl4a (ubiquitin-like protein 4a) complex reveals a novel binding interface that functions in tail-anchored protein biogenesis. The Journal of biological chemistry 290, 9387–9398, 10.1074/jbc.M114.631804 (2015). 25713138 PMC4392246

[b39] Chartron J. W., Suloway C. J., Zaslaver M. & Clemons W. M. Jr. Structural characterization of the Get4/Get5 complex and its interaction with Get3. Proceedings of the National Academy of Sciences of the United States of America 107, 12127–12132, 10.1073/pnas.1006036107 (2010). 20554915 PMC2901463

[b40] Shao S. & Hegde R. S. Target Selection during Protein Quality Control. Trends in biochemical sciences, 10.1016/j.tibs.2015.10.007 (2015). 26628391

[b41] Tanaka H. *et al.* A conserved island of BAG6/Scythe is related to ubiquitin domains and participates in short hydrophobicity recognition. FEBS J 283, 662–677, 10.1111/febs.13618 (2016). 26663859

[b42] Xu Y., Liu Y., Lee J. G. & Ye Y. A ubiquitin-like domain recruits an oligomeric chaperone to a retrotranslocation complex in endoplasmic reticulum-associated degradation. The Journal of biological chemistry 288, 18068–18076, 10.1074/jbc.M112.449199 (2013). 23665563 PMC3689951

[b43] Wang F., Whynot A., Tung M. & Denic V. The mechanism of tail-anchored protein insertion into the ER membrane. Molecular cell 43, 738–750, 10.1016/j.molcel.2011.07.020 (2011). 21835666 PMC3614002

[b44] Bielskiene K., Bagdoniene L., Mozuraitiene J., Kazbariene B. & Janulionis E. E3 ubiquitin ligases as drug targets and prognostic biomarkers in melanoma. Medicina (Kaunas) 51, 1–9, 10.1016/j.medici.2015.01.007 (2015). 25744769

[b45] Chasapis C. T. & Spyroulias G. A. RING finger E(3) ubiquitin ligases: structure and drug discovery. Curr Pharm Des 15, 3716–3731 (2009). 19925422 10.2174/138161209789271825

[b46] Sattler M., Schwendinger M. G., Schleucher J. & Griesinger C. Novel strategies for sensitivity enhancement in heteronuclear multi-dimensional NMR experiments employing pulsed field gradients. Journal of biomolecular NMR 6, 11–22, 10.1007/BF00417487 (1995). 22911576

[b47] Delaglio F. *et al.* NMRPipe: a multidimensional spectral processing system based on UNIX pipes. Journal of biomolecular NMR 6, 277–293 (1995). 8520220 10.1007/BF00197809

[b48] Skinner S. P. *et al.* Structure calculation, refinement and validation using CcpNmr Analysis. Acta crystallographica. Section D, Biological crystallography 71, 154–161, 10.1107/S1399004714026662 (2015). 25615869 PMC4304695

[b49] Shen Y., Delaglio F., Cornilescu G. & Bax A. TALOS+: a hybrid method for predicting protein backbone torsion angles from NMR chemical shifts. Journal of biomolecular NMR 44, 213–223, 10.1007/s10858-009-9333-z (2009). 19548092 PMC2726990

[b50] Rieping W. *et al.* ARIA2: automated NOE assignment and data integration in NMR structure calculation. Bioinformatics 23, 381–382, 10.1093/bioinformatics/btl589 (2007). 17121777

[b51] Koradi R., Billeter M. & Wuthrich K. MOLMOL: a program for display and analysis of macromolecular structures. J Mol Graph 14, 51–55, 29–32 (1996). 10.1016/0263-7855(96)00009-4 8744573

[b52] Kuzmic P. DynaFit–a software package for enzymology. Methods Enzymol 467, 247–280, 10.1016/S0076-6879(09)67010-5 (2009). 19897096

