# Supplementary material for: Structural and functional insights into the E3 ligase, RNF126
Source: Sci Rep. 2016 May 19;6:26433. doi: 10.1038/srep26433 (PMC4872217; doi:10.1038/srep26433)
Supplement: Supplementary Information [file srep26433-s1.docx]

**Supplementary Information**

**Structural and functional insights into the E3 ligase, RNF126**

Ewelina M. Krysztofinska^1*^, Santiago Martínez-Lumbreras^1*^, Arjun Thapaliya^1^, Nicola J. Evans^1^, Stephen High^2^ & Rivka L. Isaacson^1†^

^1^Department of Chemistry, King’s College London, Britannia House, Trinity Street, London, SE1 1DB, U.K.

^2^Faculty of Life Sciences, University of Manchester, The Michael Smith Building, Oxford Road, Manchester, M13 9PT, U.K.

^*^These authors contributed equally to the work

^†^Corresponding author; e-mail: [rivka.isaacson@kcl.ac.uk](mailto:rivka.isaacson@kcl.ac.uk) telephone: +4420 7848 7338

**Supplemental figures**


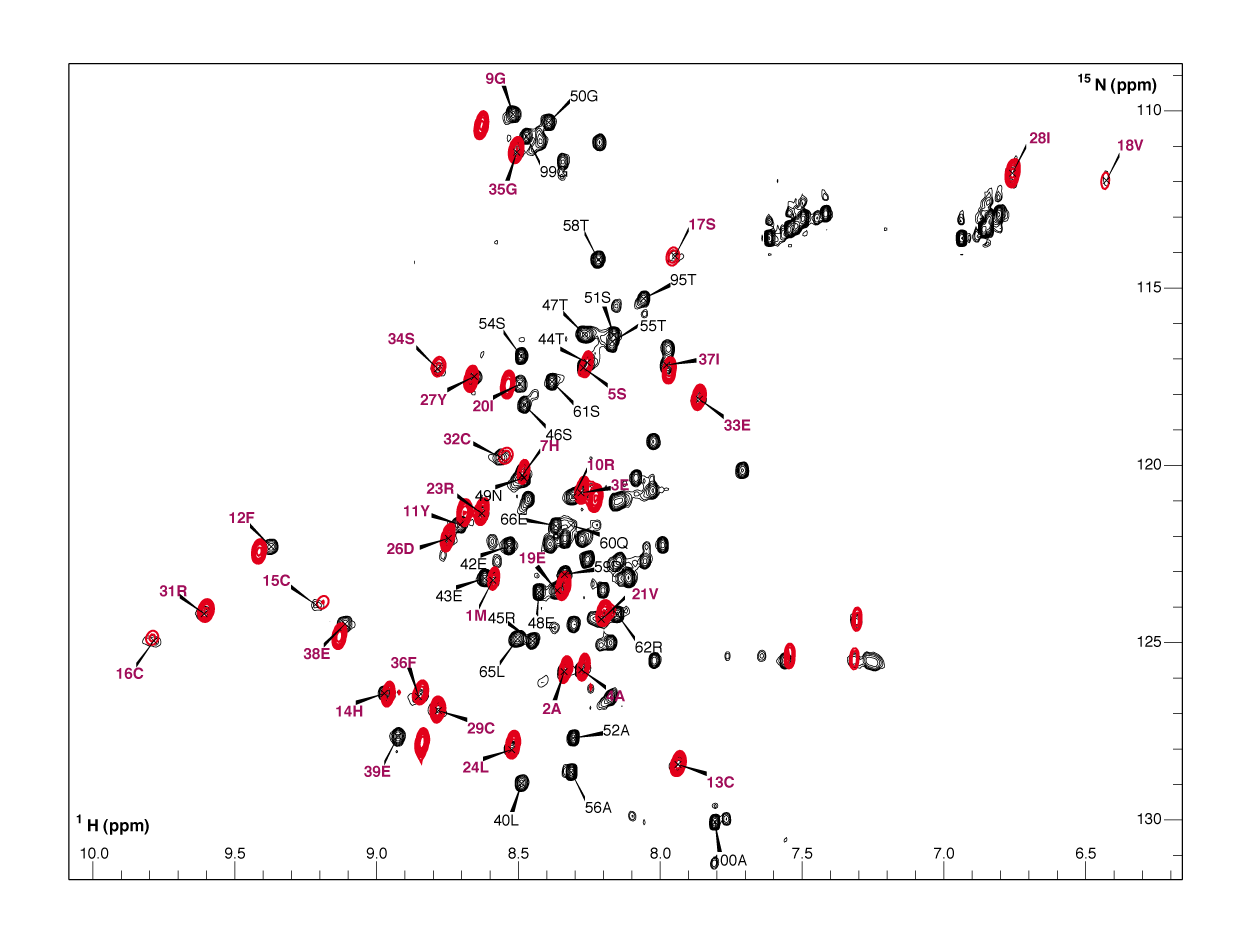


**Figure S1:** The folding of longer and shorter RNF126 constructs is conserved. ^1^H-^15^N HSQC spectra of ^15^N-labelled RNF126 (1-100) in black and the shorter version RNF126_NZF (1-40) in red. Assigned peaks from region 1-40 are labelled in red.


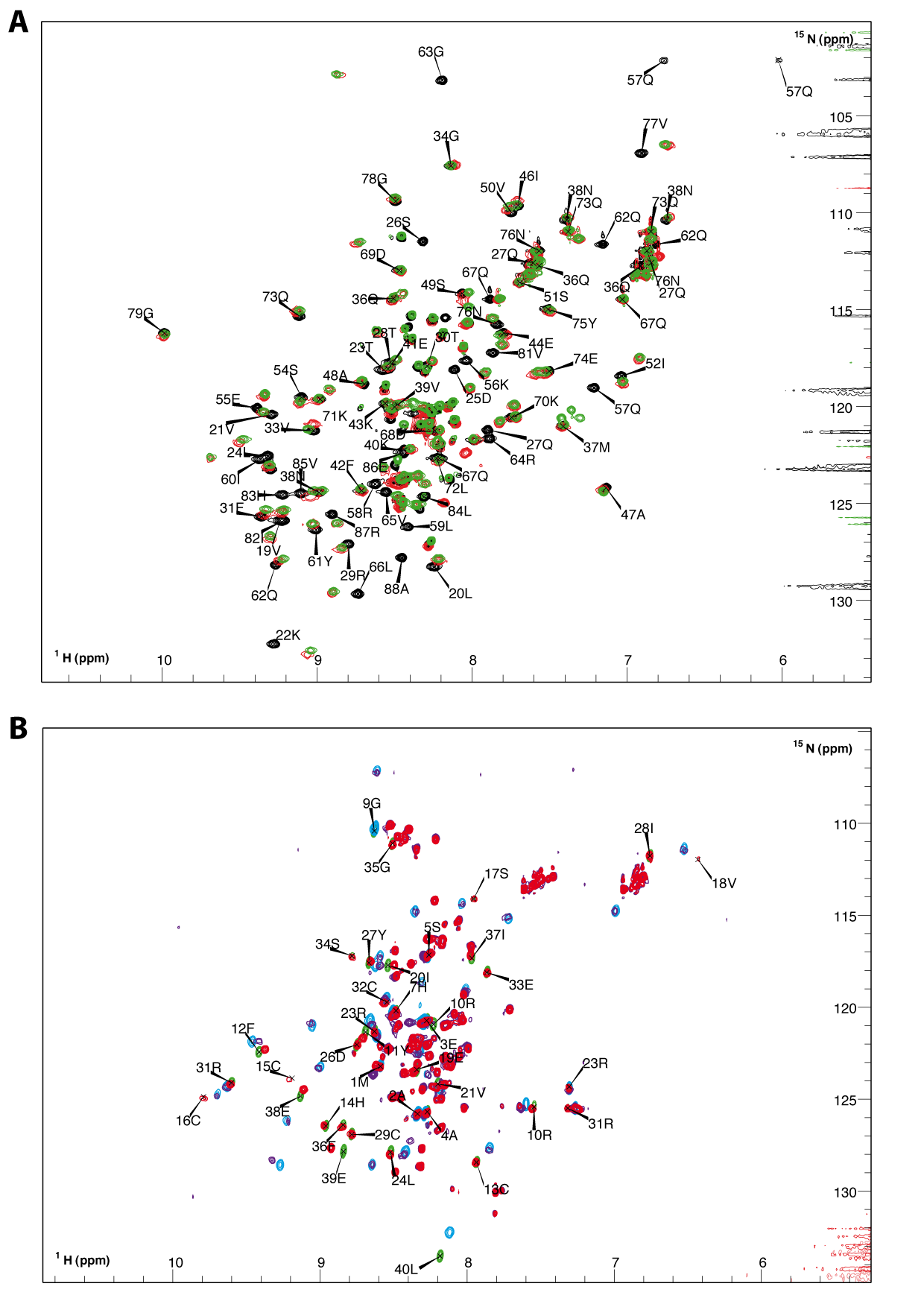


**Figure S2**: RNF126_NZF is sufficient for BAG6_UBL interaction. A) ^1^H-^15^N HSQC spectra of ^15^N-labelled BAG6_UBL in free form (black with assignments), ^15^N-labelled BAG6_UBL bound to unlabelled RNF126_NZF (1-40) at 1:1.5 molar ratio (in green) and ^15^N-labelled BAG6_UBL interacting with unlabelled RNF126_1-100 construct at the same molar ratio (in red). Bound states of BAG_UBL to both RNF126 constructs are similar. B) ^1^H-^15^N HSQC overlapping spectra of ^1^H-^15^N-labelled RNF126_NZF (green) and RNF126_1-100 (red) in free form and in bound state to unlabelled BAG6_UBL at a 1:1.5 molar ratio (NZF construct in sky blue and 1-100 in purple). Both RNF126 constructs interact in the same way with BAG6_UBL as they present the same chemical shift perturbations.


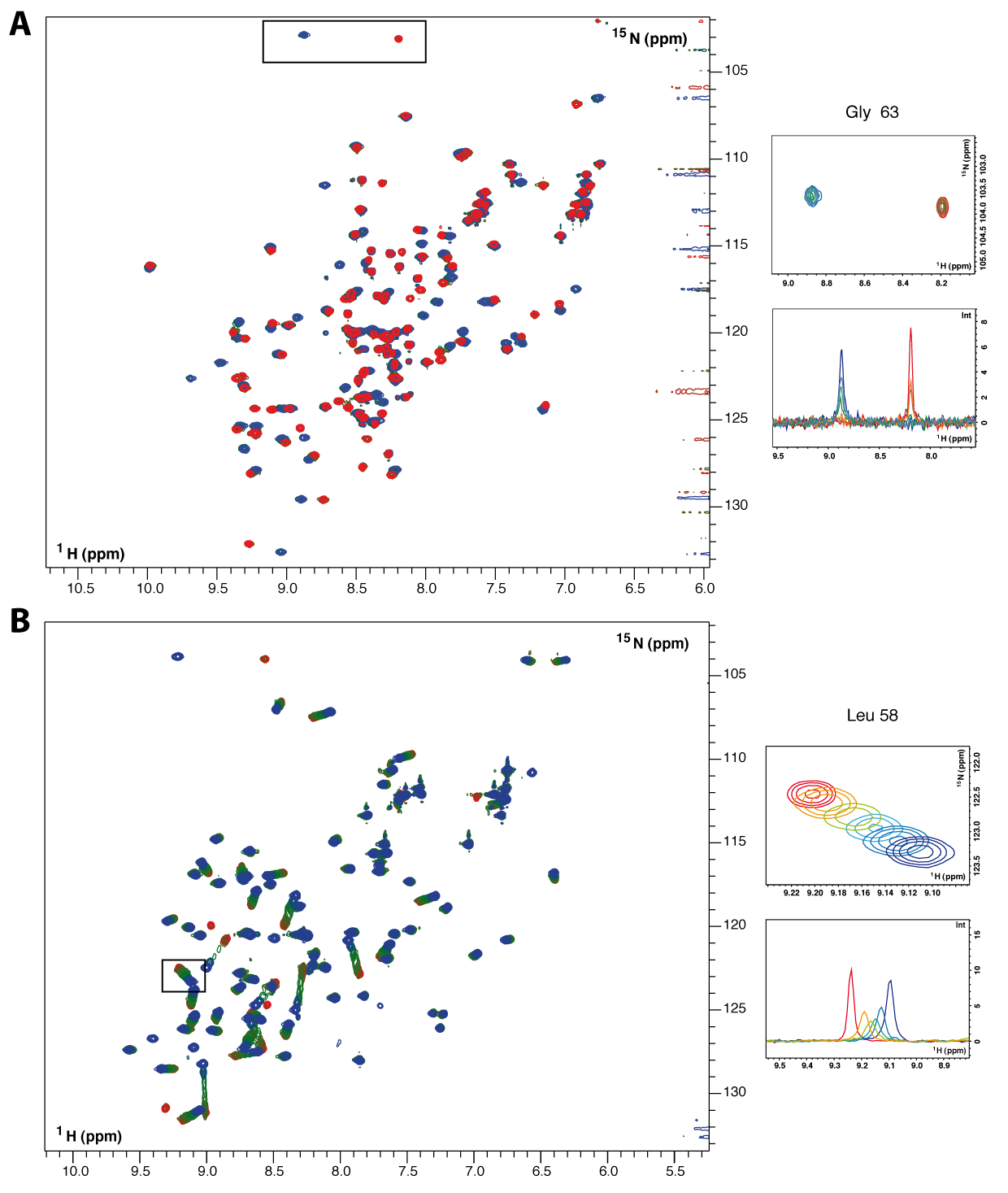


**Figure S3**: Different regimen of RNF126 binding to BAG6 (slow) and UBL4A (fast). A) ^1^H-^15^N HSQC spectra of ^15^N-labelled BAG6_UBL at different titration points with unlabelled RNF126_NZF (1:0, 1:0.25, 1:0.5, 1:0.75, 1:1, 1:2; in corresponding colours: red, orange, light green, cyan, blue, dark blue). On the right a detail of a spectra region, showing Gly63 shifting (up) and the peak intensities (down). B) Similar representation as before but in this case of ^15^N-labelled UBL4A_UBL titrated with RNF126_NZF in the same conditions. The detail on the right shows the shifting and peak intensities of Leu58 signal.


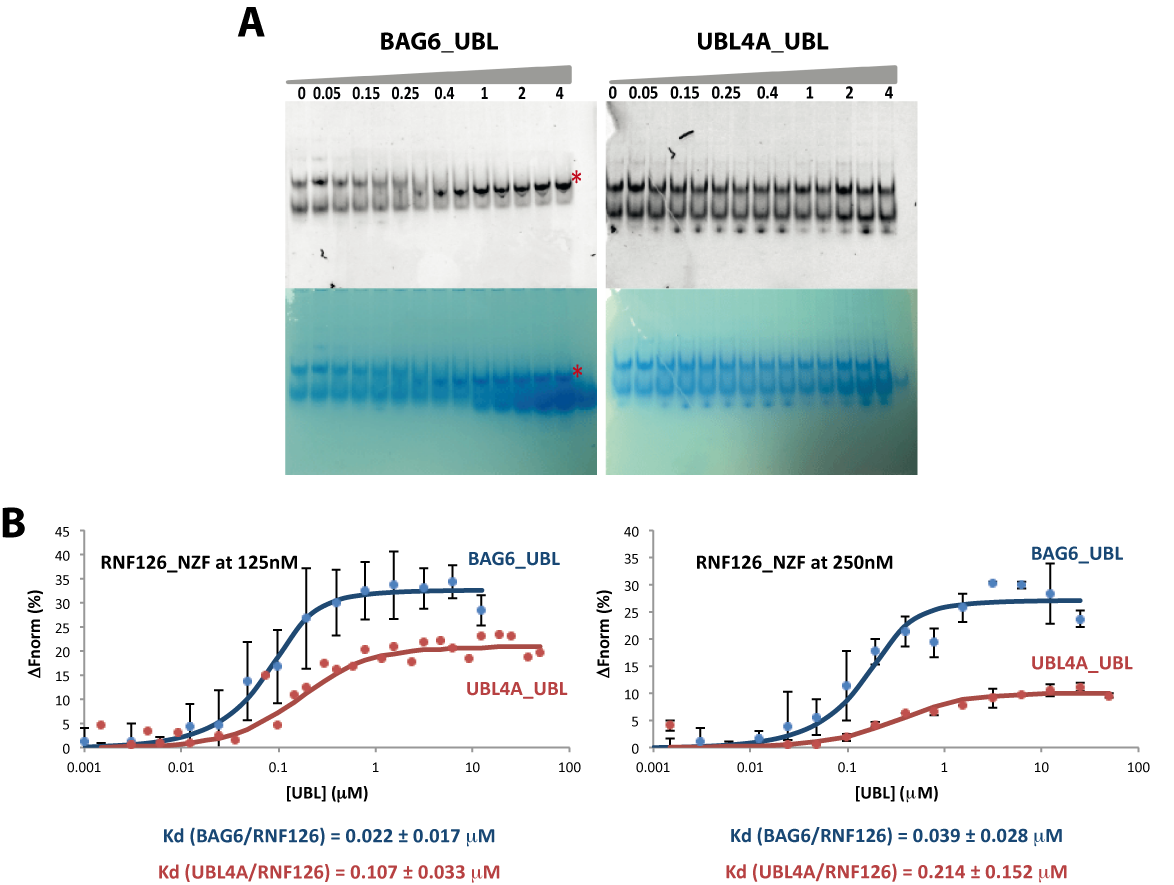


**Figure S4**: Binding of RNF126_NZF to UBL domains. A) Native PAGE of GFP tagged RNF126_NZF binding assay with UBLs (BAG6_UBL on the left, UBL4A_UBL on the right) visualised by fluorescence (top) and Coomassie (bottom). Values on the top of the gel indicate RNF126:UBL molar equivalents. The red asterisk indicates complex formation. B) MST data showing the interaction of GFP tagged RNF126_NZF at two different concentrations (0.125 and 0.250 μM) with BAG6_UBL (blue) and UBL4A_UBL (red). The change in normalized fluorescence is plotted against the concentration of unlabelled ligand and the affinity values obtained from fitting with Dynafit [52] are displayed in the figure. N.B. The Kd values derived from MST are approximately an order of magnitude lower than the values obtained by ITC indicating higher affinity. Experimental constraints necessitated using the labelled protein at a similar concentration to the expected Kd, meaning that small errors in GFP protein concentration have disproportionate impact on the results, potentially leading to overestimation of the affinity. We used 0.25 or 0.125 µM GFP protein while the Kd is 0.4 µM (ITC). We made many attempts to resolve this including using lower GFP concentrations to move away from the Kd value but we were unable to observe sufficient fluorescence signal (even after increasing the LED power). We include these results because they support the relative affinities between the different proteins. However we suspect that ITC gives us the more accurate values for Kd.


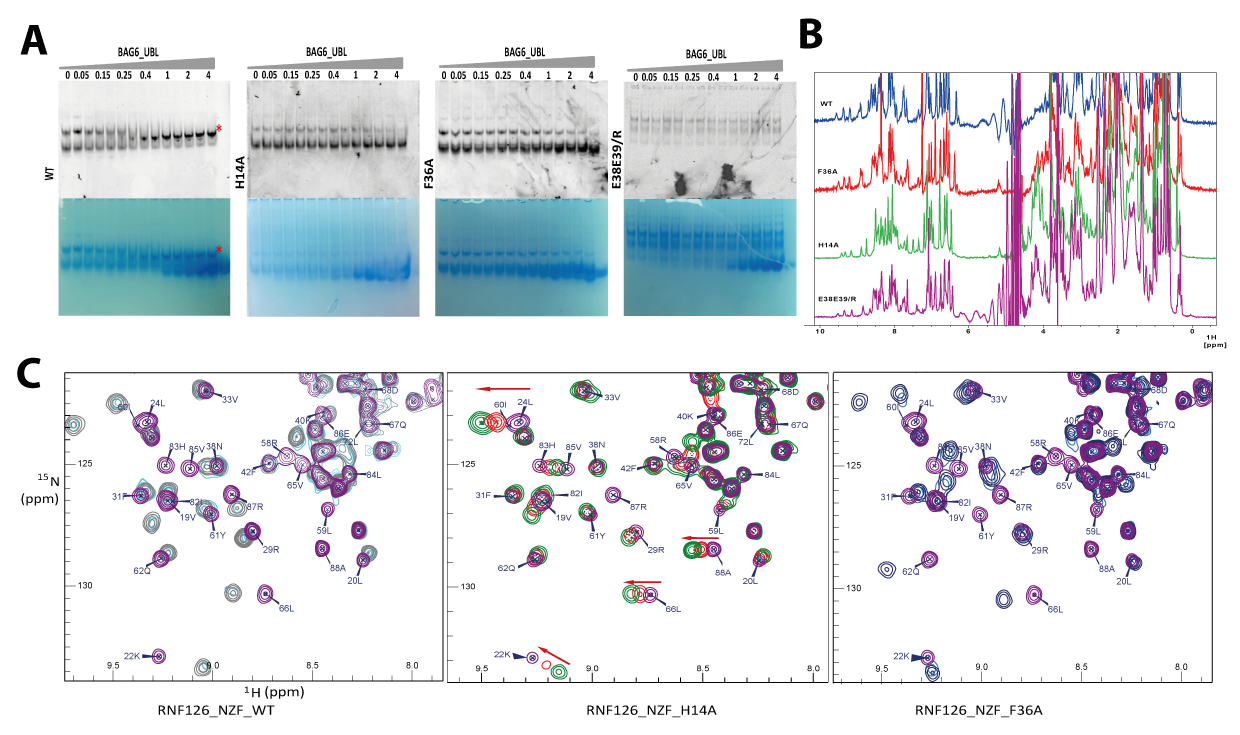


**Figure S5**: RNF126_NZF mutants present lower affinity for BAG6_UBL than wild type. A) Native PAGE of GFP-tagged RNF126_NZF_WT and mutants visualised by fluorescence (top) and Coomassie (bottom). B) 1D ^1^H NMR spectra of RNF126_NZF_WT and mutants, showing characteristic shifts of the folded protein. C) ^1^H-^15^N HSQC spectra of labelled BAG6_UBL upon titration with RNF126_NZF_WT, RNF126_NZF_H14A and RNF126_NZF_F36A. Chemical shift perturbation occupies a slow chemical exchange regimen in the case of both wild type and the F36A mutant and a fast chemical exchange regimen in the case of mutant H14A.


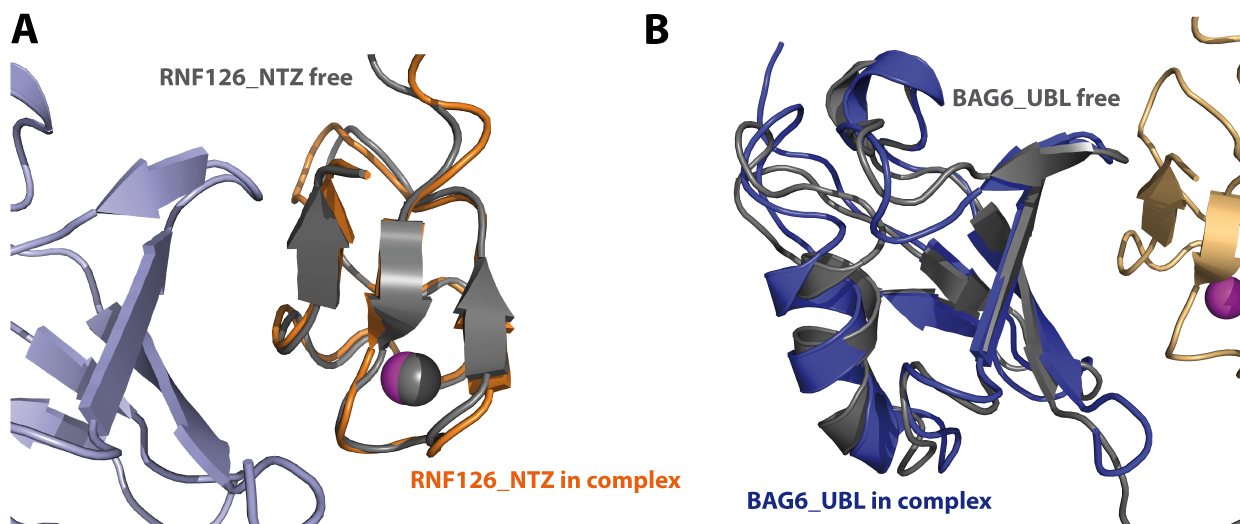


**Figure S6**: Structural alignment of RNF126_NZF (A) in free (grey, this work) and bound state (orange, this work) and structural alignment of BAG6_UBL (B) in free (grey, PDB: 1WX9) and bound state to RNF126 (blue, this work). Backbone RMSD values are: RNF126_NZF (10-40) RMSD= 0.62; BAG6_UBL (17-88) RMSD= 1.43.


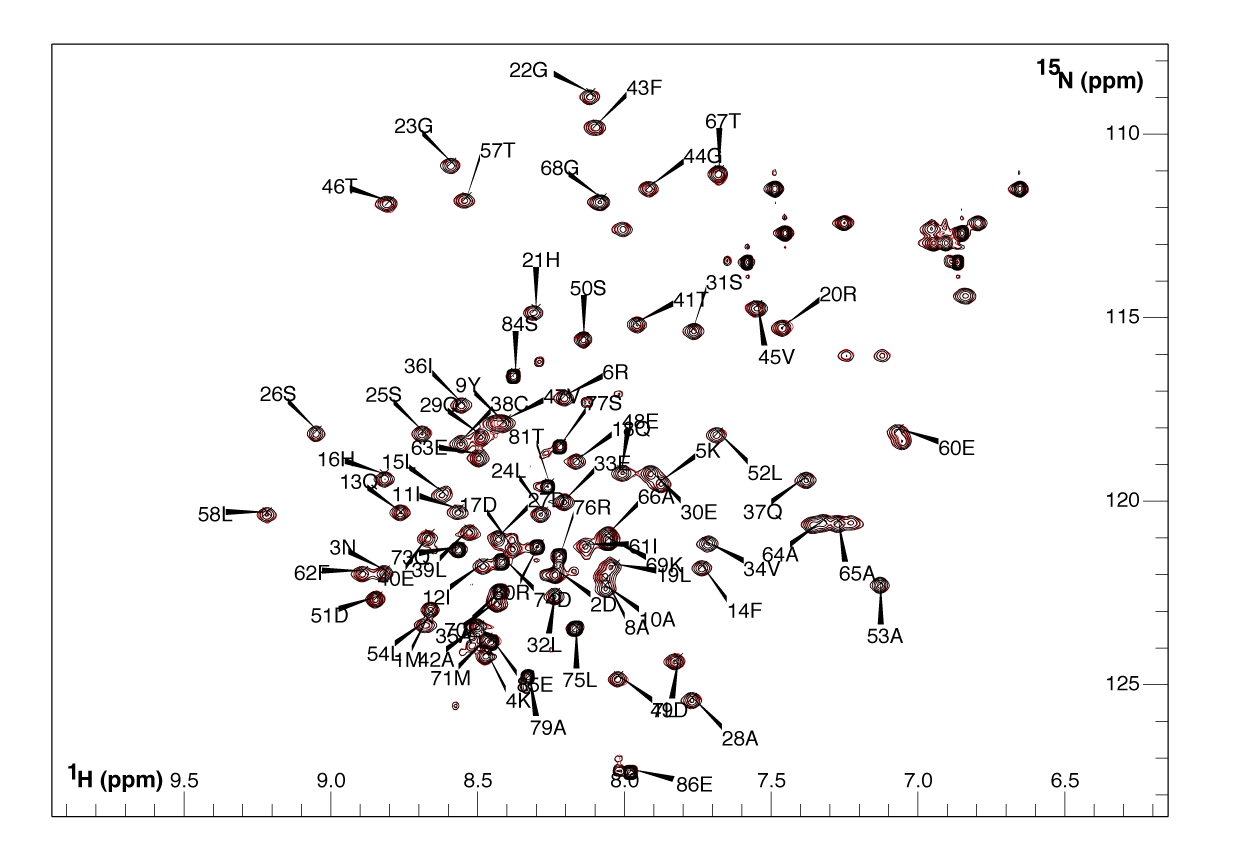


**Figure S7**: SGTA_NT and RNF126_NZF do not interact. ^1^H-^15^N HSQC spectra of ^15^N-labelled SGTA_NT dimer (assignment extracted from BMRB accession number 19779) in free form (black), and with a four-fold molar excess of unlabelled RNF126_NZF (red).


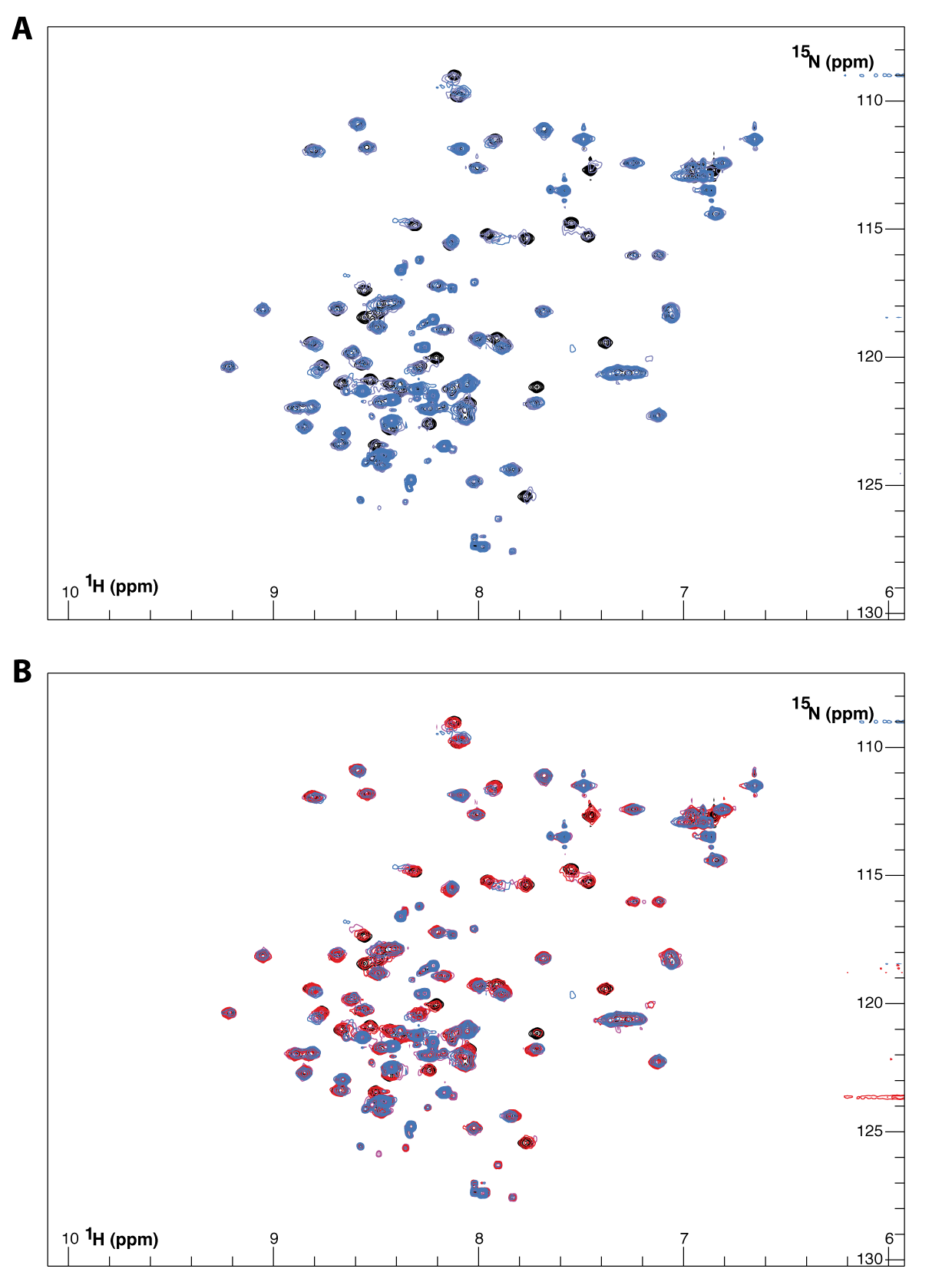


**Figure S8**: Expanded ^1^H-^15^N HSQC spectra corresponding to the detailed views in Figure 4A. A) ^15^N-labelled SGTA_NT titration with unlabelled BAG6_UBL. B) ^15^N-labelled SGTA_NT in complex with unlabelled BAG6_UBL titrated with unlabelled RNF126_NZF. Molar ratios and colours as described in the legend for Figure 4.


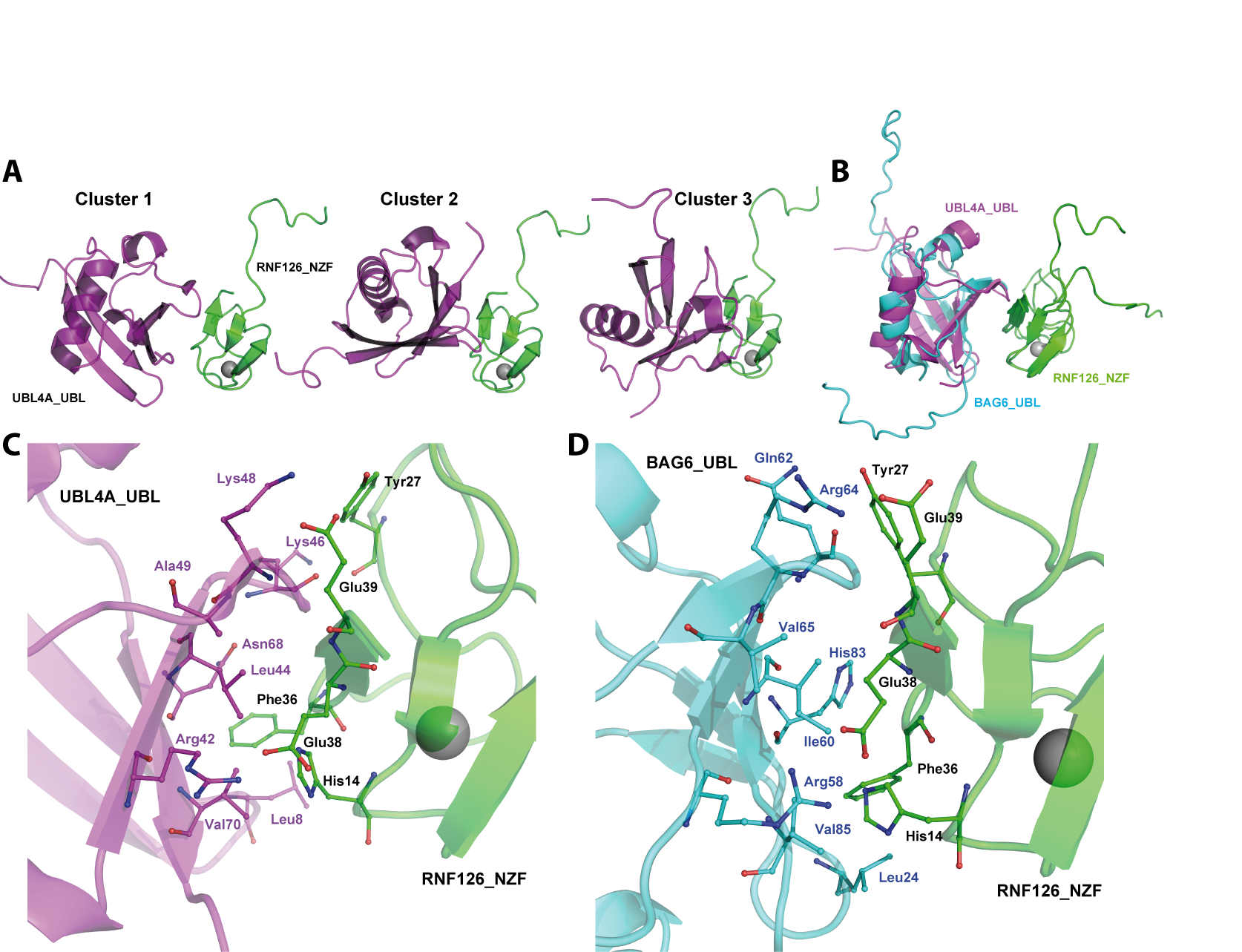


**Figure S9.** RNF126_NZF/UBL4A_UBL complex structure modelling results. A) Three top scoring clusters of the RNF126_NZF/UBL4A_UBL complex generated by HADDOCK. B) Overlay of RNF126_NZF/BAG6_UBL complex structure as calculated using ARIA with the HADDOCK-generated top scoring cluster 1 of RNF126_NZF/UBL4A_UBL complex. C) and D) Details of the binding interface of RNF126_NZF/UBL4A_UBL (C) and RNF126_NZF/BAG6_UBL (D), displaying the equivalent pertinent residues as sticks in both structures.

**
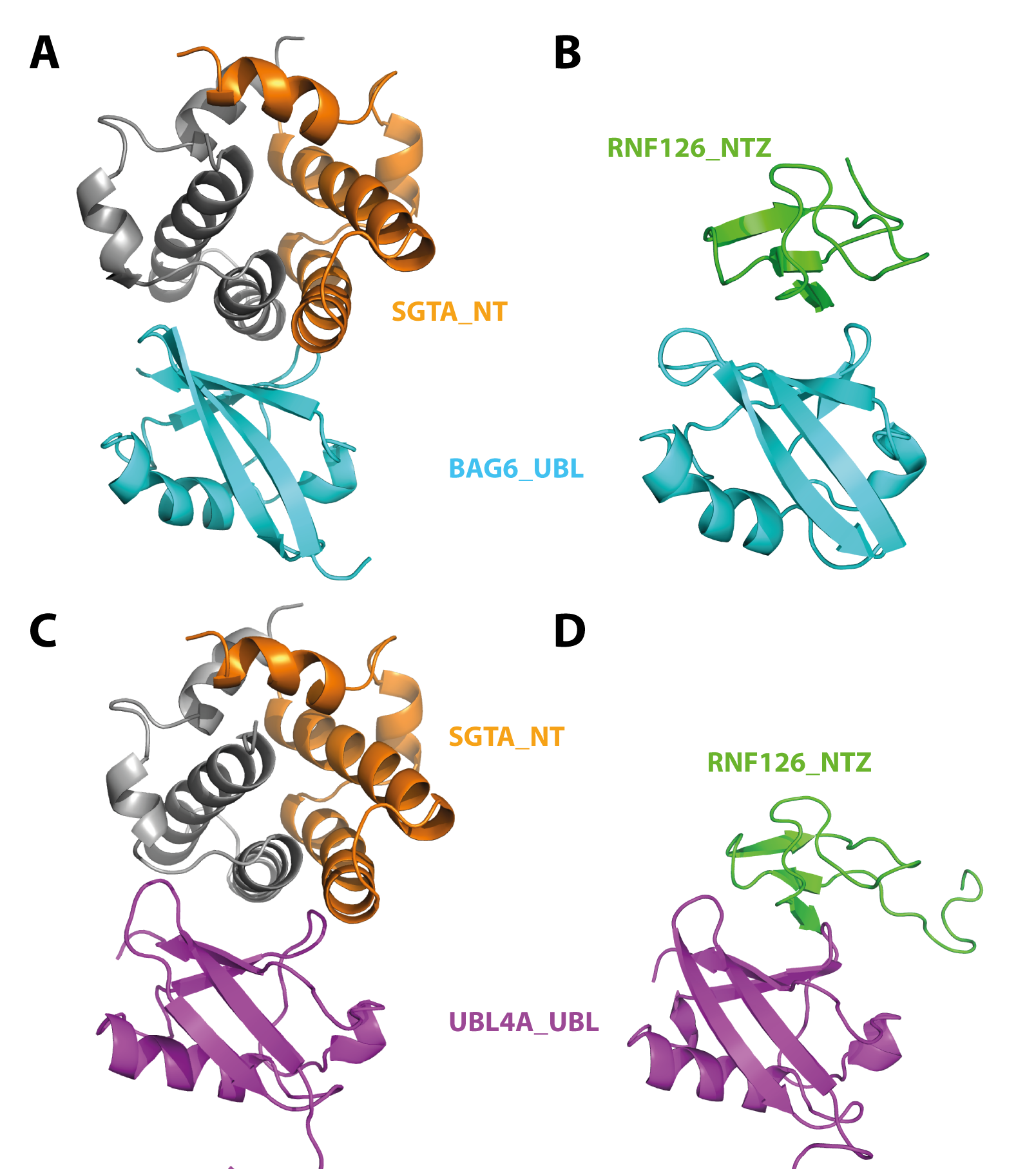
**

**Figure S10**: SGTA_NT and RNF126_NZF interact with the same region of the UBL domains of BAG6 and UBL4A. Cartoon representations of BAG6_UBL in complex with SGTA (A, HADDOCK model obtained from [31]) and with RNF126_NZF (B, solution structure, this work) and UBL4A_UBL in complex with SGTA_NT (C, HADDOCK model obtained from [31]) and RNF126_NZF (D, HADDOCK model, this work).


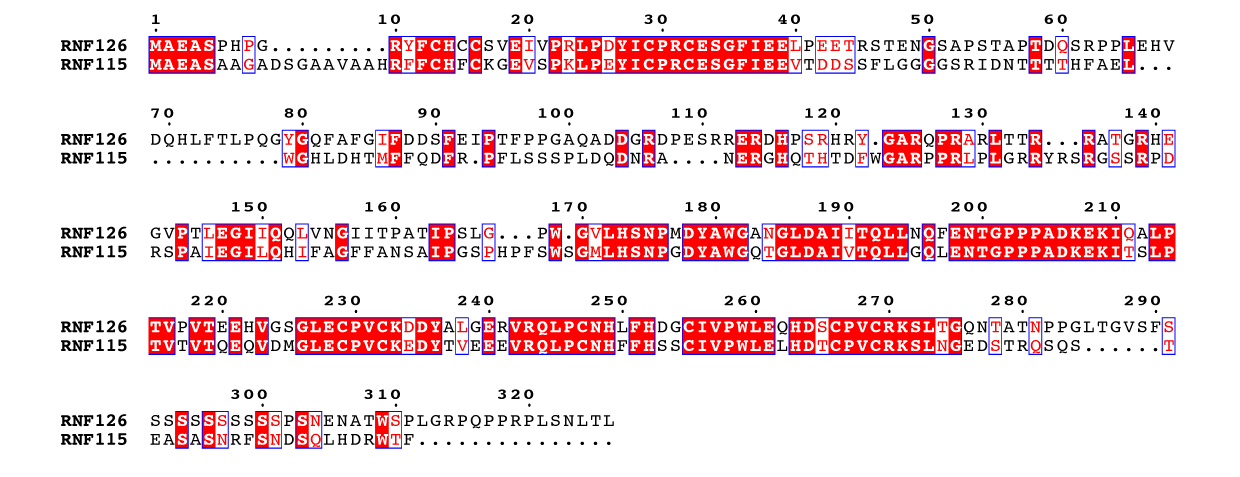


**Figure S11**: Sequence alignment of human E3 ligase proteins RNF126 and RNF115. The proteins present high conservation in the N-terminal zinc finger (10-40 of RNF126) and in a region comprising the RING domain (229-270 of RNF126). Boxes show conserved residues while red highlights sequence identity; figure generated using ESPript 3.0 server.

**Supplemental tables**

|  | Cys 13 | Cys 16 | Cys 29 | Cys 32 |
| --- | --- | --- | --- | --- |
| First coordination shell |  |  |  |  |
| Sγ-Zn distance | 2.29 ± 0.01 | 2.29 ± 0.01 | 2.29 ± 0.01 | 2.29 ± 0.01 |
| Cβ-Sγ-Zn angle | 110.0 ± 0.3 | 109.9 ± 0.3 | 105.7 ± 0.5 | 110.1 ± 0.3 |
| Second coordination shell |  |  |  |  |
| Sγ-HN (i+2) distance | 2.42 ± 0.08 | 5.24 ± 0.04 | 2.49 ± 0.04 | 3.08 ± 0.07 |
| Sγ-H-N (i+2) angle | 130.0 ± 5.3 | 91.1 ± 0.8 | 150.1 ± 1.9 | 127.3 ± 4.1 |

**Table S1**: Geometry of the zinc coordination centre of RNF126_NZF. Averaged distance (Å) and angular (º) values for first and second coordination shell across the NMR ensemble. Two clear hydrogen bonds are established in the outer shell of coordination (Cys13 and Cys29) and Cys32 is also likely.

|  | Cys 13 | Cys 16 | Cys 29 | Cys 32 |
| --- | --- | --- | --- | --- |
| First coordination shell |  |  |  |  |
| Sγ-Zn distance | 2.29 ± 0.01 | 2.29 ± 0.01 | 2.30 ± 0.01 | 2.29 ± 0.01 |
| Cβ-Sγ-Zn angle | 108.0 ± 1.5 | 110.0 ± 1.0 | 106.4 ± 1.0 | 108.9 ± 1.0 |
| Second coordination shell |  |  |  |  |
| Sγ-HN (i+2) distance | 3.02 ± 0.76 | 4.74 ± 0.48 | 2.60 ± 0.58 | 2.97 ± 0.81 |
| Sγ-H-N (i+2) angle | 122.5 ± 7.2 | 137.3 ± 6.3 | 129.3 ± 5.0 | 143.0 ± 9.1 |

**Table S2**: Geometry of the zinc coordination centre of RNF126_NZF in the RNF126_NZF/BAG6_UBL complex structure. Averaged distance (Å) and angular (º) values for first and second coordination shell across the NMR ensamble. Similar values compared with free form are obtained.

| Cluster | HADDOCK score | Cluster size | RMSD (Å) | Van der Waals energy (kcal/mol) | Restraints violation energy (kcal/mol) | Buried surface área (Å^2^) | Electrostatic energy (kcal/mol) | Desolvation energy (kcal/mol) | Z score |
| --- | --- | --- | --- | --- | --- | --- | --- | --- | --- |
| Cluster1 | -91.7 ± 2.7 | 119 | 1.5 ± 1.2 | -32.9 ± 7.4 | 5.0 ± 2.0 | 1017.7 ± 74.4 | -297.3 ± 64.4 | 0.2 ± 4.0 | -1.3 |
| Cluster2 | -79.1 ± 3.6 | 23 | 5.9 ± 0.8 | -31.3 ± 5.4 | 3.3 ± 1.8 | 1031.1 ± 98.6 | -302.9 ± 21.8 | 12.5 ± 2.8 | -0.7 |
| Cluster3 | -73.9 ± 6.7 | 21 | 9.7 ± 0.8 | -25.0 ± 3.0 | 16.1 ± 17.4 | 958.2 ± 30.2 | -274.5 ± 23.9 | 4.4 ± 3.0 | -0.4 |

**Table S3**: Energetic parameters obtained for the three best clusters of RNF126_NZF/UBL4A_UBL complex calculated using HADDOCK.
